# Islet Autoantibody Standardization Program: interlaboratory comparison of insulin autoantibody assay performance in 2018 and 2020 workshops

**DOI:** 10.1007/s00125-023-05877-9

**Published:** 2023-02-10

**Authors:** Ilaria Marzinotto, David L. Pittman, Alistair J. K. Williams, Anna E. Long, Peter Achenbach, Michael Schlosser, Beena Akolkar, William E. Winter, Vito Lampasona, participating laboratories

**Affiliations:** 1 San Raffaele Diabetes Research Institute, San Raffaele Scientific Institute, Milan, Italy; 2 Department of Pathology, University of Florida, Gainesville, FL USA; 3 Diabetes and Metabolism, Translational Health Sciences, University of Bristol, Bristol, UK; 4 Institute of Diabetes Research, Helmholtz Munich, German Research Center for Environmental Health, Neuherberg, Germany; 5 Department of General Surgery, Visceral, Thoracic and Vascular Surgery, University Medical Center Greifswald, Greifswald, Germany; 6 Institute of Pathophysiology, Research Group of Predictive Diagnostics, University Medical Center Greifswald, Karlsburg, Germany; 7 Division of Diabetes, Endocrinology, and Metabolic Diseases, National Institute of Diabetes and Digestive and Kidney Diseases, Bethesda, MD USA

**Keywords:** Autoantibodies, IAA, IASP interlaboratory comparison study, Sensitivity, Specificity, Type 1 diabetes

## Abstract

**Aims/hypothesis:**

The Islet Autoantibody Standardization Program (IASP) aims to improve the performance of immunoassays measuring autoantibodies in type 1 diabetes and the concordance of results across laboratories. IASP organises international workshops distributing anonymised serum samples to participating laboratories and centralises the collection and analysis of results. In this report, we describe the results of assays measuring IAA submitted to the IASP 2018 and 2020 workshops.

**Methods:**

The IASP distributed uniquely coded sera from individuals with new-onset type 1 diabetes, multiple islet autoantibody-positive individuals, and diabetes-free blood donors in both 2018 and 2020. Serial dilutions of the anti-insulin mouse monoclonal antibody HUI-018 were also included. Sensitivity, specificity, area under the receiver operating characteristic curve (ROC-AUC), partial ROC-AUC at 95% specificity (pAUC95) and concordance of qualitative/quantitative results were compared across assays.

**Results:**

Results from 45 IAA assays of seven different formats and from 37 IAA assays of six different formats were submitted to the IASP in 2018 and 2020, respectively. The median ROC-AUC was 0.736 (IQR 0.617–0.803) and 0.790 (IQR 0.730–0.836), while the median pAUC95 was 0.016 (IQR 0.004–0.021) and 0.023 (IQR 0.014–0.026) in the 2018 and 2020 workshops, respectively. Assays largely differed in AUC (IASP 2018 range 0.232–0.874; IASP 2020 range 0.379–0.924) and pAUC95 (IASP 2018 and IASP 2020 range 0–0.032).

**Conclusions/interpretation:**

Assay formats submitted to this study showed heterogeneous performance. Despite the high variability across laboratories, the in-house radiobinding assay (RBA) remains the gold standard for IAA measurement. However, novel non-radioactive IAA immunoassays showed a good performance and, if further improved, might be considered valid alternatives to RBAs.

**Graphical abstract:**

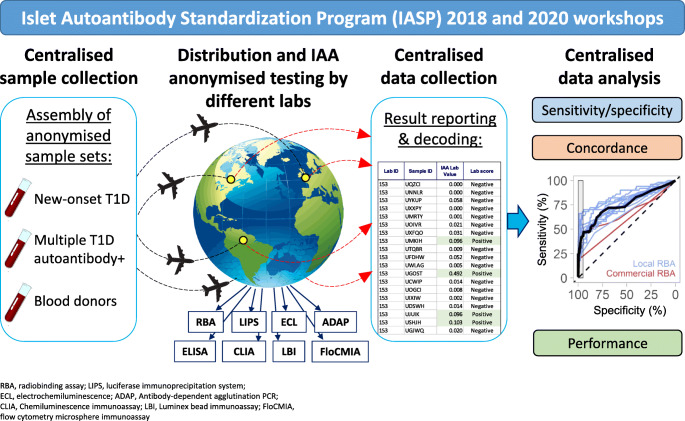

**Supplementary Information:**

The online version contains peer-reviewed but unedited supplementary material available at 10.1007/s00125-023-05877-9.

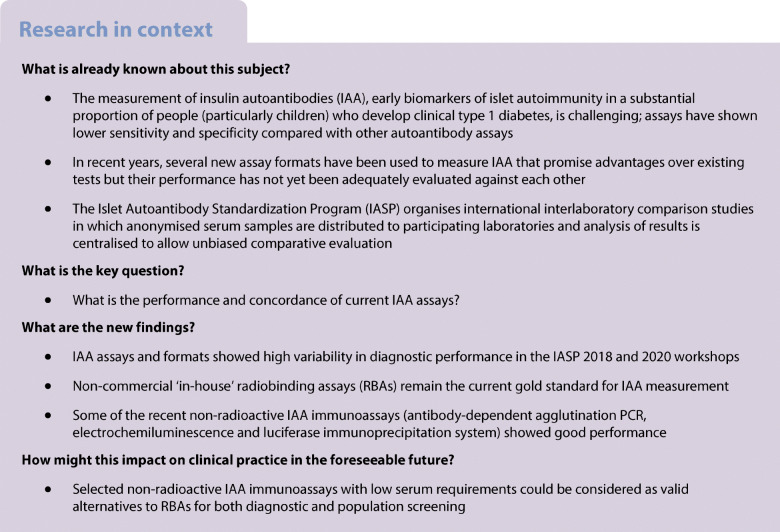



## Introduction

Antibody reactivity to insulin was first described in individuals undergoing exogenous insulin administration [1] but people with different autoimmune diseases can also produce IAA in the absence of prior treatment with the hormone. IAA were first described in the insulin autoimmune syndrome (IAS, Hirata’s disease) in 1970 [2] and then in type 1 diabetes in 1983 [3]. The development of insulin autoimmune hypoglycaemia has been linked to exposure to environmental triggers (e.g. drugs and food supplements) in individuals with IAS [4], while no environmental factors have been confirmed in type 1 diabetes.

IAA are among the first biomarkers to appear during type 1 diabetes natural history. In asymptomatic individuals at risk of diabetes, the appearance of IAA associates with a faster progression to overt type 1 diabetes and younger age at onset [5, 6]. Additionally, specific features of IAA, such as binding affinity, targeted epitopes and titre, are associated with a higher risk of rapid type 1 diabetes development [7–9]. For these reasons, the measurement of IAA has become a cornerstone of screening strategies for type 1 diabetes [10–13] and a particular focus has been given to the improvement of IAA assays.

The first attempts at the standardisation of IAA measurement highlighted a large variability of results across centres and assays [14–18]. More recent interlaboratory comparisons studies organised by the Diabetes Autoantibody Standardization Program (DASP) showed only partial improvements in assay performance and results concordance [19, 20].

The Islet Autoantibody Standardization Program (IASP) has superseded the DASP in promoting the continuous improvement of type 1 diabetes autoantibody assays and disseminating empirically tested best-practice protocols, state-of-the-art reagents and serum standards [21]. The IASP is a collaborative effort supported by the Immunology of Diabetes Society (IDS) and the US NIH, which is run by the University of Florida Pathology Laboratories, Endocrine Autoantibody Laboratory and coordinated by an IDS nominated committee. The IASP pursues its goals through the establishment of a periodic interlaboratory comparison of type 1 diabetes-associated autoantibody measurements, aimed at providing an unbiased assessment of assay performance and improving the concordance of results across laboratories around the world. In IASP workshops the participating laboratories test type 1 diabetes autoantibodies in anonymised type 1 diabetes patient, ‘at-risk’ person, and control serum samples. An unbiased comparison of assay performance is provided through the centralised collection and analysis of results by the IASP committee.

In this report, we present the results of the 2018 and 2020 IASP IAA assays interlaboratory comparison studies that were preliminarily presented at the IASP 2018 and IASP 2020 workshops, held at the 16th and 17th IDS Congress, respectively.

## Methods

### Study design

The study was aimed at comparing assay performance across laboratories. Participating laboratories received the same sera in anonymised sets, each labelled with an aliquot-specific unique code. Sera were obtained from the following individuals: individuals with new-onset type 1 diabetes (contributed by several centres around the world), collected within 14 days of the first insulin treatment; multiple islet autoantibody-positive first-degree relatives (FDR) of individuals with type 1 diabetes (enrolled in the TrialNet Ancillary Study – Pathway to Prevention and showing a transiently altered GTT during screening); blood donors without diabetes, collected in the USA. Due to their heterogeneous origin and to the difficulty of procuring sufficiently large serum volume from young children, these samples were only partially representative of incident new-onset diabetes and age-matched control individuals.

All samples were collected upon written informed consent, with the approval of local ethics committees and according to the ethical principles for medical research involving human subjects of the Declaration of Helsinki [22].

In 2018, the set included samples from 43 individuals with new-onset diabetes, seven multiple islet autoantibody-positive FDRs, 90 blood donor control individuals, six serial dilutions (156, 20, 5, 1.2, 0.6 and 0 ng/ml) in normal human serum of HUI-018, an IgG1 anti-insulin mouse monoclonal antibody (mAb) targeting a conformational epitope spanning the insulin A and B chains [23], and four additional samples from non-diseased individuals with type 1 diabetes autoantibodies.

Individuals with type 1 diabetes had a median age of 14 years (range 8–47) and included 15 female individuals and 28 male individuals; 37 were White, two Black, two of mixed ancestry and two of undisclosed ancestry. The FDRs had a median age of 16 years (range 12–53) and included four female and three male individuals, all of White ancestry. Demographic data were available only for 88 non-diabetic blood donors, who had a median age of 20 years (range 18–30), and included 44 female and 44 male individuals, of whom 69 were White and 19 Black.

In 2020, the set included samples from 38 individuals with new-onset diabetes, 12 multiple islet autoantibody-positive FDRs, 90 control blood donors, HUI-018 mAb serial dilutions, and four standards from the National Institute of Diabetes and Digestive and Kidney Diseases (NIDDK) consortium, corresponding to 235, 5.8, 2 and 0 DK units, made from a mixture of GADA- and IA-2A-positive sera and used as reference material to harmonise antibody assays [24].

Individuals with type 1 diabetes had a median age of 14 years (range 8–47) and included 15 female and 23 male individuals, of whom 31 were White, two Hispanic, three Black, one of mixed ancestry and one of undisclosed ancestry. FDRs had a median age of 18 years (range 10–53) and included seven female and five male individuals, of whom 11 were White and one was of mixed ancestry.

Only a minority of samples were present in both 2018 and 2020 sample sets (21 type 1 diabetes, six FDRs, one blood donor) (electronic supplementary material [ESM] Table 1).

### Data analysis

Laboratory personnel were asked to report details of their assay protocol, assay raw data and results using uniform Excel reporting sheets. All data analyses were performed in the R language and environment for statistical computing and graphics [25].

Assays sensitivity and specificity was calculated as the percentage of case (new-onset plus FDRs) sera reported as IAA positive and as the percentage of blood donor sera reported as IAA-negative, respectively. Adjusted sensitivity at 95% (AS95), 99% (AS99) and 100% specificity (AS100) were calculated after placing the threshold for positivity at the 95th, 99th and 100th percentiles of values observed in the blood donor samples in each assay, respectively.

Concordance of laboratory-assigned positive or negative scores across assays was expressed as average pairwise per cent agreement (APPA) between assays (i.e. the average number of times each possible combination of two assays agreed on IAA-positive/negative scores divided by the number of samples scored). We tested the occurrence of agreement by pure chance by calculating Gwet’s coefficient of inter-rater agreement reliability (AC1) [26] and Fleiss’ coefficient of inter-rater agreement reliability (*k*) [27] using the irr R package [28].

Assay performance in discriminating case from control samples was analysed using the area under the receiver operating characteristic curve (ROC-AUC) and the partial ROC-AUC at 95% specificity (pAUC95) [29] using the pROC R package [30].

Interassay antibody titre concordance was analysed by calculating Kendall’s ranking agreement coefficient (W) [31], after ranking of case and control samples according to autoantibody levels in each assay. The significance of differences in mean ranking of selected case samples between different assays was tested using the Mann–Whitney test.

In a subset of assays with good performance (pAUC95 ≥0.015) and an immune-complex capture system compatible with the measurement of an anti-mouse IgG mAb, local units were converted into common units (HUI-018 ng/ml equivalents) by applying a linear model to the local units attributed to the provided HUI-018 dilutions followed by rescaling of local quantitative results. The concordance of antibody units was then evaluated by calculating the overall concordance correlation coefficient (OCCC) according to Barnhart using the epiR R package [32].

For all statistical analyses, two-tailed *p* values <0.05 were considered as significant.

## Results

### Summary of submitted IAA assay formats in the IASP 2018 and IASP 2020 workshops

**Fig. 1:**
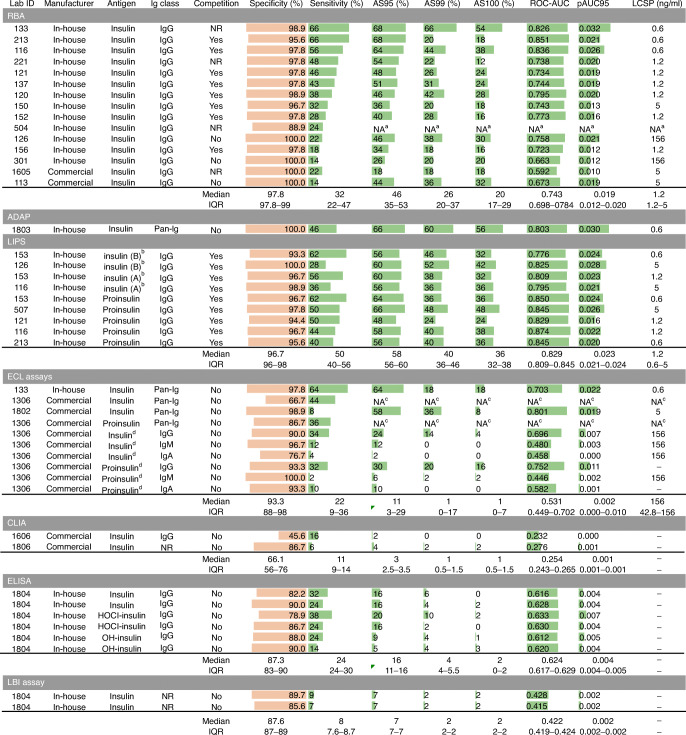
IASP 2018 assay formats: main characteristics and performance. Single assays and their main characteristics are reported with the corresponding specificity (orange bars indicate the % specificity, with a maximum of 100%), sensitivity, AS95, AS99, AS100 (green bars indicate the % sensitivity, with a maximum of 100%), ROC-AUC, pAUC95 (green bars indicate the maximum value of 1 and 0.05, respectively) and LCSP. Assays are grouped by format. The median and the IQR values of each variable are reported for each group. ^a^Local units were not reported by the participating laboratory (only positivity scores). ^b^The NanoLuc reporter is alternatively placed near the insulin B or A chain. ^c^Positivity scores in pan-Ig ECL assays for laboratory 1306 were assigned based on the combination (and/or) of positivity scores in the corresponding multiplexed Ig class-specific ECL assays (thus, no units were reported for either assay). ^d^Multiplexed Ig class-specific insulin or proinsulin assays. NR, not reported

In the IASP 2018 workshop, 23 laboratories from 13 countries submitted results from 45 IAA assays. The breakdown of assays according to format was as follows: radiobinding assay (RBA) [33]; antibody-dependent agglutination PCR (ADAP) [34]; luciferase immunoprecipitation system (LIPS) [35]; electrochemiluminescence (ECL) [36]; chemiluminescence immunoassay (CLIA); ELISA assay (of which some measured antibodies to oxidised insulin [37]); and Luminex bead immunoassay (LBI) (Fig. 1).

All RBAs used recombinant insulin radiolabelled with ^125^I. LIPS assays used proinsulin (*n*=5) or insulin antigens (*n*=4) with the NanoLuc luciferase reporter tagged either at the C-terminus of the B chain ([pro]insulin-B-NLuc) or the N-terminus of the A chain (insulin-A-NLuc). Most RBA and LIPS assays were competitive assays performed with or without the presence of untagged insulin competitor (5.6 × 10^−6^ mol/l and 3.6 × 10^−7^ mol/l, respectively).

Antigen–antibody binding occurred in liquid phase in 35 assays (ADAP, ECL, LIPS, RBA) and was followed by the capture of immune complexes via recovery of immunoglobulins (LIPS, RBA) or tagged antigen (ADAP, ECL). Antigen–antibody binding occurred in hybrid solid/liquid phase in two assays (CLIA) and in solid phase for eight (ELISA, LBI). Major characteristics and metrics of each individual assay are reported in ESM Table 2.

**Fig. 2:**
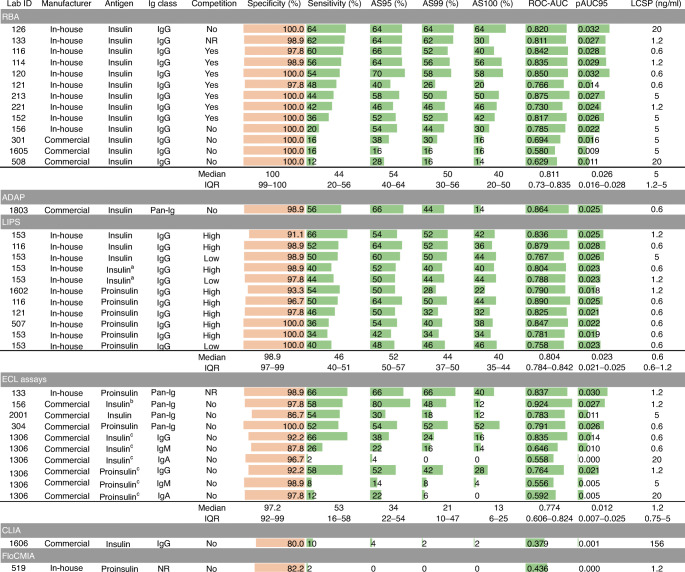
IASP 2020 assay formats: main characteristics and performance. Single assays and their main characteristics are reported with the corresponding specificity (orange bars indicate the % specificity, with a maximum of 100%), sensitivity, AS95, AS99, AS100 (green bars indicate the % sensitivity, with a maximum of 100%), ROC-AUC, pAUC95 (green bars indicate the maximum value of 1 and 0.05, respectively) and LCSP. Assays are grouped by format. The median and the IQR values of each variable are reported for each group. ^a^Duplex LIPS assays multiplexing IAA and IA-2A testing using a dual luciferase system. ^b^Multiplexed with the measurement of IA-2A, GADA, ZnT8A and autoantibodies to transglutaminase (TGA). ^c^Multiplexed Ig class-specific insulin or proinsulin assays. NR, not reported

In the IASP 2020 workshop, 22 laboratories from 11 countries submitted results from 37 IAA assays (Fig. 2) based on the following formats: RBA; ADAP; LIPS; ECL; CLIA; and Flow cytometric microsphere-based immunoassay (FloCMIA) [38].

All RBAs used recombinant insulin radiolabelled with ^125^I. LIPS assays used either proinsulin (*n*=6) or insulin (*n*=5) antigens tagged with a NanoLuc luciferase reporter at the C-terminus of the insulin B chain. One LIPS assay (Duplex LIPS) combined individual measurement of IA-2A and IAA using a dual luciferase system. One laboratory used two different concentrations of competitor in LIPS (3.6 × 10^−7^ mol/l and 1.1 × 10^−9^ mol/l).

Antigen–antibody binding occurred in liquid phase in 36 assays (ADAP, ECL, FloCMIA, LIPS, RBA) or in hybrid solid/liquid phase (CLIA) and was followed by the capture of immune complexes through the recovery of immunoglobulins (LIPS, RBA) or antigens with different tags (ADAP, ECL, FloCMIA, CLIA). Major characteristics and metrics of each individual assay are reported in ESM Table 3.

### Assay sensitivity and specificity based on laboratory-assigned scores in the IASP 2018 and IASP 2020 workshops

In the IAA assays submitted to the IASP 2018 workshop, laboratory-assigned scores showed a median assay sensitivity of 32.0% (IQR 16.0–46.0) and a specificity of 96.7% (IQR 89.7–97.8), with a wide range for both (sensitivity 66.0–2.0%; specificity 100.0–45.6%) (Fig. 1, ESM Table 2 and ESM Fig. 1).

In the assays submitted to the IASP 2020 workshop, the median assay sensitivity was 46.0% (IQR 26.0–56.0) and the specificity 98.9% (IQR 96.7–100.0), with a wide range of both (sensitivity 66.0–2.0%; specificity 100.0–80.0%) (Fig. 2, ESM Table 3 and ESM Fig. 2).

### ROC-AUC analysis of assay format performance in the IASP 2018 and IASP 2020 workshops

**Fig. 3:**
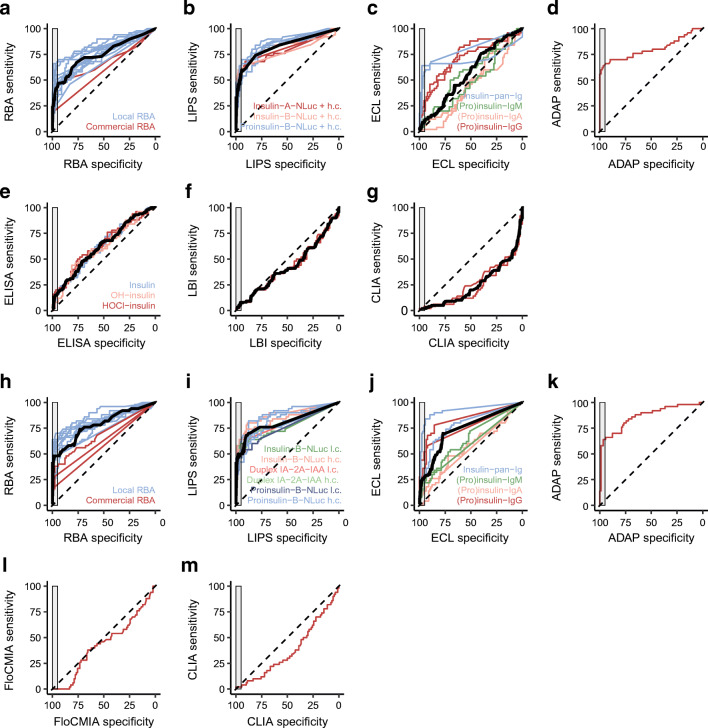
ROC curve analysis of assays submitted to the IASP 2018 (**a**–**g**) and IASP 2020 (**h**–**m**) workshops. ROC curves are shown for RBA (**a**: *n*=14 and **h**: *n*=13), LIPS (**b**: *n*=9 and **i**: *n*=11), ECL (**c**: *n*=8 and **j**: *n*=8), ADAP (**d**: *n*=1 and **k**: *n*=1), ELISA (**e**: *n*=6), LBI (**f**: *n*=2), CLIA (**g**: *n*=2 and **m**: *n*=1) and FloCMIA (**l**: *n*=1); indicated assay variants within each format are shown by curve colour. Black lines, median ROC curve; grey rectangles, area corresponding to a specificity ≥95%, where the pAUC95 is calculated; dashed lines, identity line. h.c., high concentration of unlabelled insulin competitor (3.6 × 10^−7^ mol/l); l.c., low concentration of unlabelled insulin competitor (1.1 × 10^−9^ mol/l)

We evaluated the performance of the IAA assays using the full ROC-AUC and the partial ROC-AUC after imposing a specificity of ≥95%, as a more relevant proxy of assay performance [29] (Fig. 3).

**Fig. 4:**
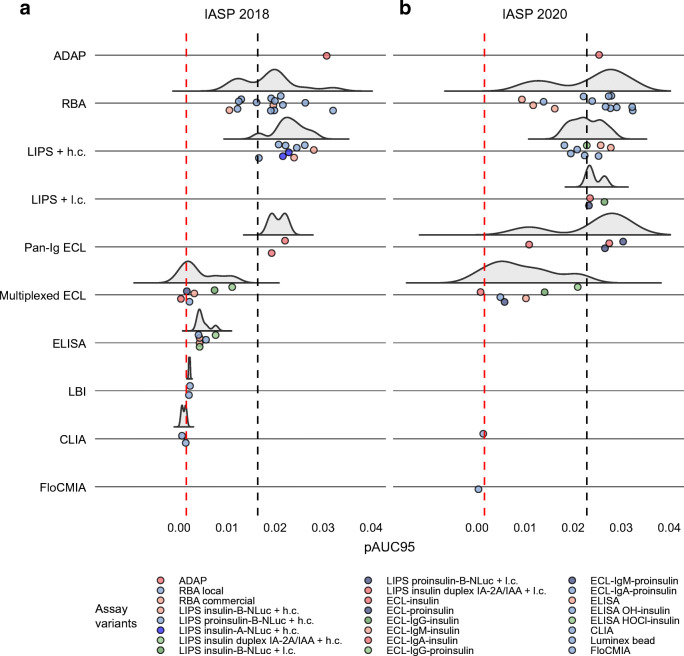
Distribution of the pAUC95 of IAA assays submitted to the IASP 2018 (**a**) and IASP 2020 (**b**) workshops. Results are grouped by assay format. LIPS assays using two alternative amounts of unlabelled insulin are labelled as either high (h.c., 3.6 × 10^−7^ mol/l) or low concentration (l.c., 1.1 × 10^−9^ mol/l). The grey half violin plots show the overall probability density estimate. Circles correspond to the pAUC95 value of each single assay, with colours indicating different assay variants. The vertical dashed lines correspond to the median pAUC95 of all assays (black) and to the ROC identity line (red), respectively

In the IASP 2018 workshop, IAA assays showed a median ROC-AUC of 0.736 (IQR 0.617–0.803, range 0.232–0.874) and a median pAUC95 of 0.016 (IQR 0.004–0.021, range 0–0.032), against a theoretical pAUC95 maximum of 0.05 (Figs 1, 4 and ESM Fig. 3a). A wide heterogeneity of performance was present both within and across assay formats.

In the IASP 2020 assays, the median ROC-AUC of the IAA assays was 0.790 (IQR 0.730–0.836, range 0.378–0.924), while the median pAUC95 was 0.023 (IQR 0.014–0.026, range 0–0.032) (Figs 2, 4 and ESM Fig. 3b). Additionally, in the IASP 2020 workshop, assay performance varied widely both within and across formats.

### Qualitative concordance of laboratory-assigned positive/negative scores

In the assays submitted to the IASP 2018 workshop, the APPA of positive/negative scores assigned to type 1 diabetes cases across all assays of IAA was 67.1%, while the first-order AC1 of these scores was 0.415. The concordance analysis was greater in control samples (APPA 86.5%, AC1 0.863). When the analysis was limited to assays with a pAUC95 above the median (pAUC95 ≥0.013), these concordance variables improved in both case (APPA 73.4%; AC1 0.476) and control samples (APPA 96.1%; AC1 0.959).

In the assays submitted to the IASP 2020 workshop, in cases the APPA was 66.9% and the AC1 0.360, while in controls the respective values were 93.7% and 0.932. In assays with a pAUC95 above the median (pAUC95 ≥0.023), these values improved in both case (APPA 74.8%; AC1 0.497) and control samples (APPA 97.3%; AC1 0.972).

In both years, the observed low Fleiss’ *k* concordance coefficients were consistent, with most of the control samples scored IAA positive being sporadically so in only a small fraction of assays.

Overall, the concordance of IAA-positive/-negative scores was greater across assays using the same format (ESM Tables 4–7).

### Assay format-specific patterns of IAA recognition

**Fig. 5:**
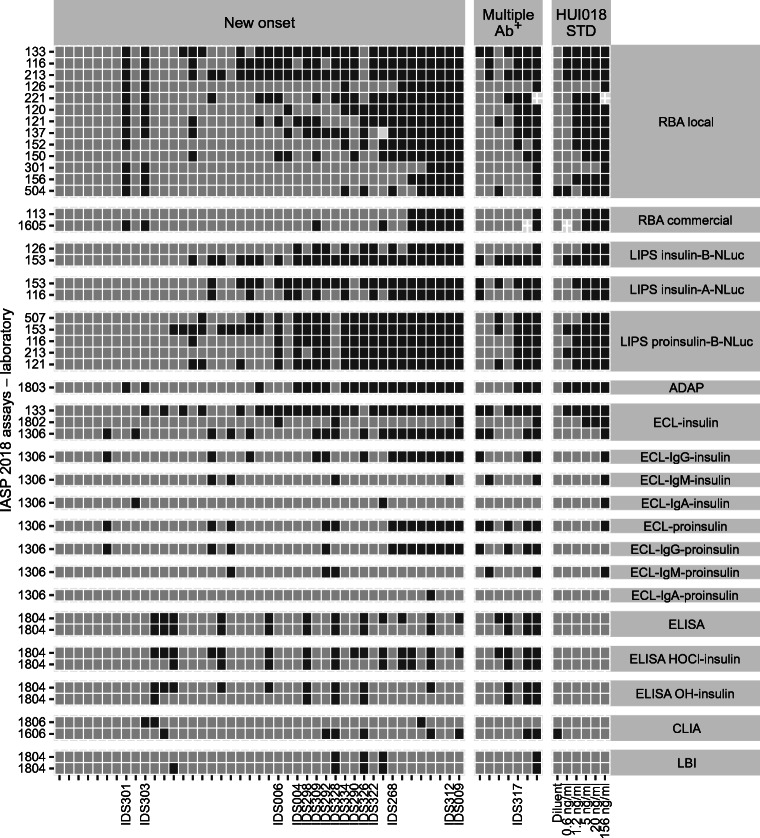
Tilemap of IAA positivity scores in case sera submitted to the IASP 2018 workshop. Tilemap of IAA-positive (dark grey) or -negative (light grey) scores assigned by laboratories to each new-onset type 1 diabetes, multiple autoantibody-positive and HUI-018 standard samples. Samples are sorted on the *x*-axis according to the median rank calculated in each group. The shown sample labels identify sera with format-specific patterns of reactivity described in the text. Assays on the *y*-axis are grouped by format or format variant and then sorted according to their median pAUC95. Ab^+^, autoantibody-positive; HUI-018 STD, HUI-018 standard dilutions

**Fig. 6:**
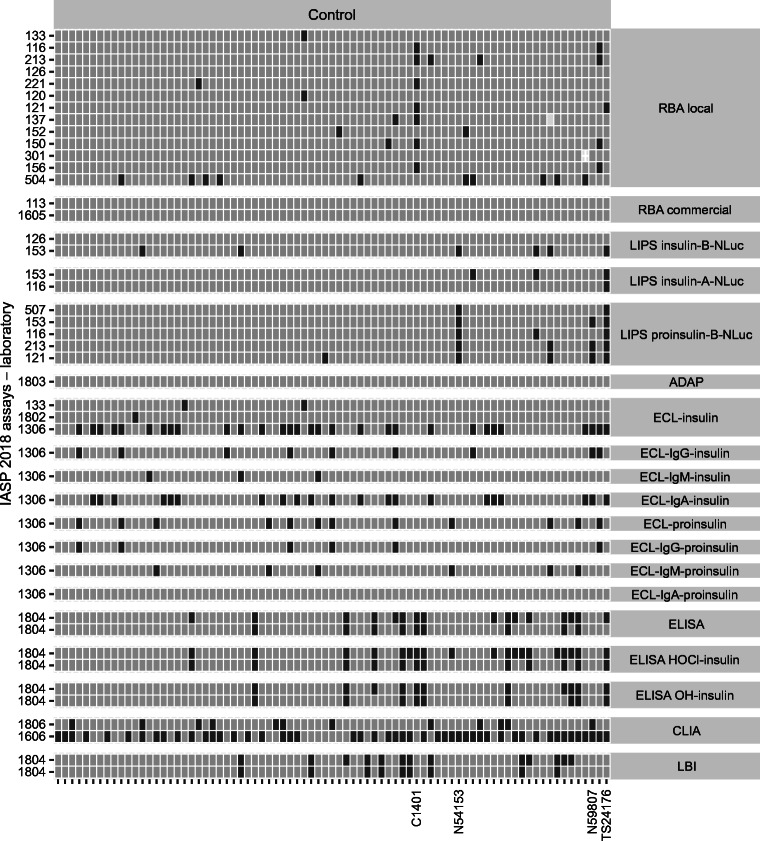
Tilemap of IAA positivity scores in control sera submitted to the IASP 2018 workshop. Tilemap of IAA-positive (dark grey) or -negative (light grey) scores assigned by laboratories to each control sample. Samples are sorted on the *x*-axis according to the median rank calculated in each group. The shown sample labels identify sera with format-specific patterns of reactivity described in the text. Assays on the *y*-axis are grouped by format or format variant and then sorted according to their median pAUC95. Only samples with a positive score in at least one assay are shown

In both IASP workshops, discrepancies in positive/negative scores showed format-specific patterns. In 2018, a subset of type 1 diabetes cases (IDS326, IDS322, IDS292, IDS298, IDS004, IDS309, IDS006, IDS290) was IAA positive predominantly in ADAP and LIPS and in few RBA and ECL assays. Conversely, a different subset of sera (IDS303, IDS301) was IAA positive in ADAP and most RBAs but only in a minority of LIPS and ECL assays. A subset of control sera (TS24176, N59807, N54153) was IAA positive mostly in LIPS and sporadically in other formats, while another control sample (C1401) was positive mostly in RBA and ELISA (Figs 5, 6 and ESM Figs 4, 5).

**Fig. 7:**
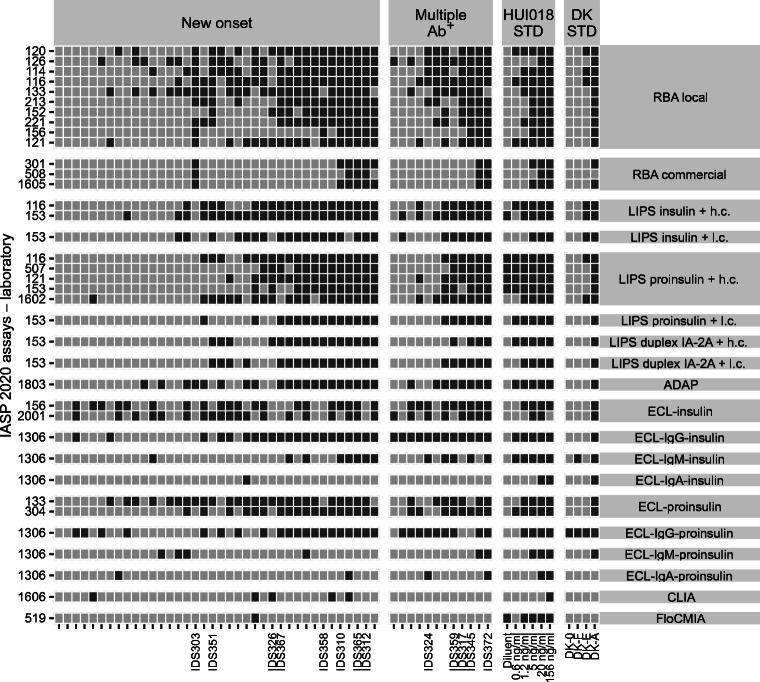
Tilemap of IAA positivity scores in case sera submitted to the IASP 2020 workshop. Tilemap of IAA-positive (dark grey) or -negative (light grey) scores assigned by laboratories to each sample of new-onset type 1 diabetes, multiple autoantibody-positive, DK standard and HUI-018 standard sera. The shown sample labels identify sera with format-specific patterns of reactivity described in the text. Samples are sorted on the *x*-axis according to the median rank calculated in each group. Assays on the *y*-axis are grouped by format or format variant and then sorted according to their median pAUC95. LIPS assays using two alternative amounts of unlabelled insulin are labelled as either high (h.c., 3.6 × 10^−7^ mol/l) or low concentration (l.c., 1.1 × 10^−9^ mol/l). Ab^+^, autoantibody-positive. DK STD, test standards distributed by the NIDDK consortium

**Fig. 8:**
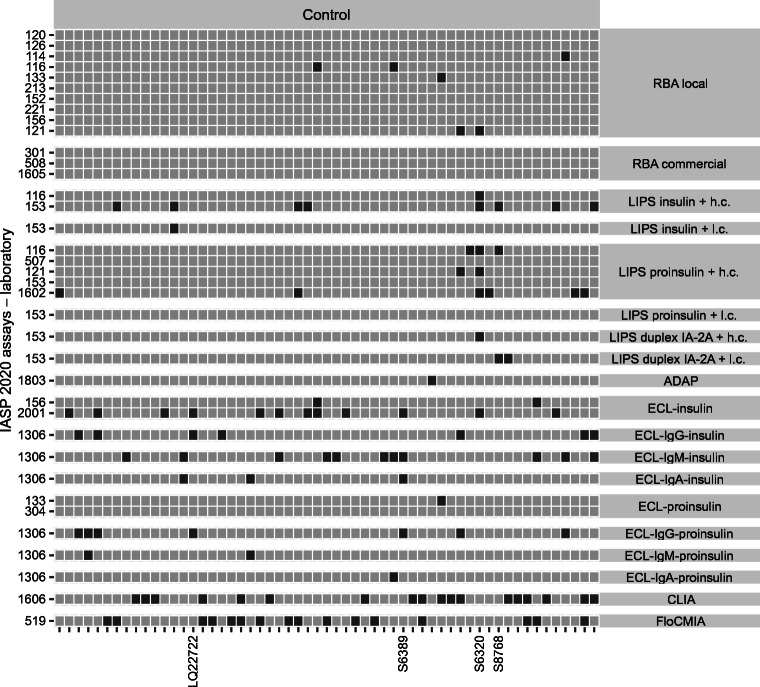
Tilemap of IAA positivity scores in control sera submitted to the IASP 2020 workshop. Tilemap of IAA-positive (dark grey) or -negative (light grey) scores assigned by laboratories to each control sample. Samples are sorted on the *x*-axis according to the median rank calculated in each group. The shown sample labels identify sera with format-specific patterns of reactivity described in the text. Assays on the *y*-axis are grouped by format or format variant and then sorted according to their median pAUC95. LIPS assays using two alternative amounts of unlabelled insulin are labelled as either high (h.c., 3.6 × 10^−7^ mol/l) or low concentration (l.c., 1.1 × 10^−9^ mol/l)

In the sera submitted to the IASP 2020 workshop, two case samples (IDS359, IDS326) were IAA positive predominantly in LIPS but only in a minority of RBA and ECL assays. Conversely, another subset of case samples (IDS324, IDS303 and IDS351) was positive in most RBA and ECL but only in a minority of LIPS assays. Among control sera, two (S6320, S8768) were positive mostly in LIPS but only sporadically in other formats, while two other serum samples (S6389, LQ22722) were positive exclusively in ECL. In LIPS assays using two different concentrations of unlabelled insulin competitor, IAA positivity was sometimes conditional upon the use of a high concentration of competitor (IDS326), suggesting the presence of low-affinity IAA. Always in LIPS, one serum sample (IDS351) resulted positive only when insulin antigen was used, suggesting that antigen recognition was conditional on removal of the C-peptide (Figs 7, 8 and ESM Figs 6, 7).

The results also highlighted the selective recognition of some case sera by assays in which antibody–antigen binding occurs in liquid phase (ADAP, ECL, LIPS, RBA) vs solid phase (CLIA, ELISA, FloCMIA, LBI) in both IASP 2018 (IDS312, IDS009, IDS268, IDS328, IDS334, IDS317) and IASP 2020 (IDS372, IDS345, IDS317, IDS312, IDS365, IDS310, IDS358, IDS367) workshops.

### Ranking of autoantibody levels

Quantitative interassay concordance of IAA levels was evaluated by ranking sera in each assay and then calculating the Kendall’s W ranks agreement coefficient.

In the IASP 2018 workshop, the W coefficient across all assays was 0.354 for case sera and 0.081 for control sera. In the IASP 2020 workshop, the W coefficient across all assays was 0.458 for case sera and 0.047 for control sera.

In both workshops, excluding assays with low performance (i.e. low pAUC95) from the analysis led to a modest increase of the agreement coefficient in both case and control samples (IASP 2018, W=0.431 and 0.086, respectively; IASP 2020, W=0.587 and 0.057, respectively). Limiting the analysis to assays with higher performance (i.e. greater than the median pAUC95) showed a further increase of W for case sera but only a marginal improvement for control sera (IASP 2018, W=0.656 and 0.098, respectively; IASP 2020, W=0.617 and 0.086, respectively).

Concordance of IAA level ranks increased among assays using the same format for both case and control sera (ESM Figs 8–15 and ESM Tables 4–7). The comparison of assay formats’ W coefficients showed that ranking agreement was higher in LIPS than in both local and commercial RBAs, CLIA and ECL assays.

IAA level ranking also confirmed the previously observed preferential recognition of some case samples by assays in which antibody–antigen binding occurs in liquid phase vs solid phase (Mann–Whitney test, all *p*≤0.001 in IASP 2018 workshop and all *p*<0.05 in IASP 2020 workshop).

### Autoantibody levels in 2018 vs 2020

Using the 28 samples that were distributed in both the IASP 2018 workshop and the IASP 2020 workshop, we analysed the correlation of quantitative results in 24 assays (nine local RBAs, one commercial RBA, one ADAP, five LIPS, one CLIA and seven ECL). Correlation was highest in RBA, LIPS and ADAP (median *R*^2^=0.97 [IQR 0.94–0.98]) and lower in ECL and CLIA (median *R*^2^=0.25 [IQR 0.01–0.50]) (ESM Figs 16, 17).

### Conversion of local units into common HUI-018 units

Using the provided HUI-018 anti-insulin mAb serial dilutions, we converted local arbitrary units into common units (HUI-018 ng/ml) by applying a log–log linear regression model. In both the IASP 2018 and the IASP 2020 workshop, the correlation of local and common units was high (*R*^2^ range 0.85–1.00) but the slopes of the regression curves varied considerably across assays (slope range 0.406–1.000) (ESM Figs 18, 19). We then compared calculated common HUI-018 units in each assay with the true concentration of the HUI-018 serial dilutions (ESM Figs 20, 21). The observed high degree of variability of the assigned concentrations was suggestive of potentially large discrepancies in the linear range of the different assays.

### Autoantibody levels in common HUI-018 units

**Fig. 9:**
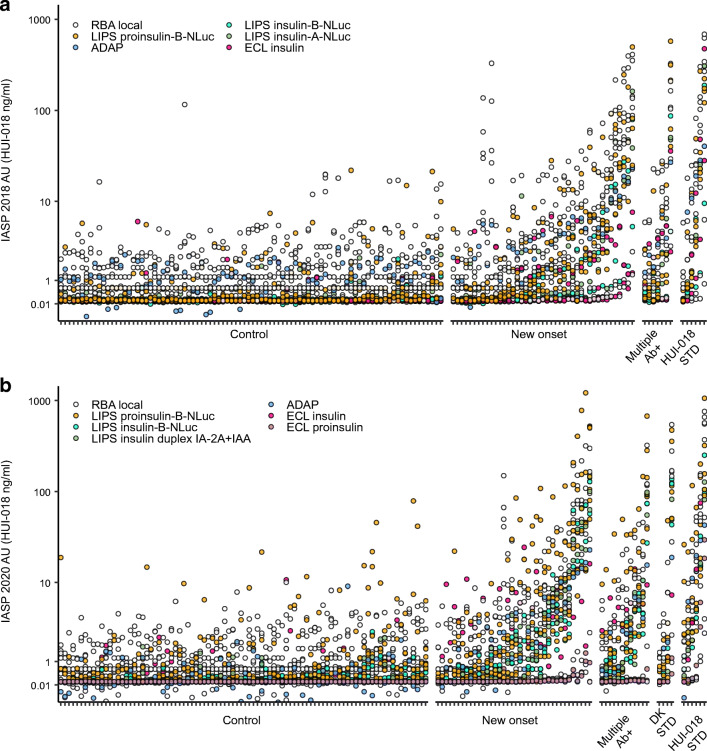
Stripchart of common HUI-018 units in selected assays submitted to the IASP 2018 (**a**) and IASP 2020 (**b**) workshops. Samples from blood donors, individuals with new-onset type 1 diabetes, multiple autoantibody positivity, DK and HUI-018 standards are sorted on the *x*-axis according to the median rank calculated in each group. Circles show the HUI-018 units attributed to each sample; circle colour indicates assay format. Ab^+^, autoantibody-positive; AU, arbitrary units calculated as HUI-018 ng/ml equivalents. DK STD, test standards distributed by the NIDDK consortium

We converted local arbitrary units into common HUI-018 units for assays with good performance and a detection system compatible with the measurement of a mouse IgG mAb (Fig. 9). We then calculated the OCCC of HUI-018 units as another measure of interassay quantitative concordance. In both workshops, the OCCC was relatively low in both case (IASP 2018, 0.285; IASP 2020, 0.203) and control sera assays (IASP 2018, 0.013; IASP 2020, 0.012). After stratification of assays according to format and antigen, the quantitative concordance of common units improved only for some assays (ESM Figs 22, 23 and ESM Tables 4–7).

### Analytical sensitivity of IAA assays

As a proxy of the IAA assays’ analytical sensitivity, we determined the lowest concentration scored positive (LCSP) by each assay for HUI-018 mAb (Figs 1, 2, 5, 7). The median LCSP was 1.2 ng/ml in both workshops but exhibited extreme variability across assays (range 0.6–156). In addition, some assays recognised the diluent normal serum as weakly positive.

## Discussion

Early interlaboratory comparison studies demonstrated that the detection of disease-specific IAA was crucially dependent on assay format choice and led to the establishment of the liquid-phase immunoprecipitation RBA assay as the de facto gold standard for IAA measurement [14–20].

The more recent IASP 2018 and IASP 2020 interlaboratory IAA measurement comparison studies saw not only the continued implementation of RBAs by most laboratories but also an increasingly wider adoption of alternative non-radioactive formats. Therefore, in this paper we were able to perform a comprehensive comparison of RBAs and other assay formats in terms of diagnostic performance, sensitivity, specificity and concordance.

RBAs submitted to the IASP workshop in both 2018 and 2020 were predominantly in-house assays derived from the IAA micro-assay originally described in 1997 by Williams et al [33]. The performance of these micro-assays varied widely in both workshops but compared with more recent non-radioactive IAA immunoassays most RBAs showed similar or better sensitivity and specificity, as well as pAUC95 and AS95, two metrics aimed at excluding regions of low assay specificity and poor clinical relevance from the analysis. Of note, commercial RBAs, while showing good specificity, had a sensitivity less than half that of most in-house micro-assays, possibly because of a lower capacity to immuno-precipitate IAA immune complexes by the anti-human IgG polyclonal antibody used in commercial kits compared with the protein A/G coated Sepharose beads adopted by the micro-assay RBA.

Among non-radioactive IAA immunoassays, the only submitted ADAP assay, the most recently developed IAA assay format, showed the highest or second-highest pAUC95 and AS95.

In both workshops, among non-radioactive assay formats, the most widely adopted were LIPS and ECL. Submitted LIPS assays implemented a variety of alternative protocols and antigens and showed variable performance. Nevertheless, LIPS assays showed the overall second-highest median pAUC95 and AS95 after RBA.

ECL assays comprised two major variants, the first measuring IAA potentially of any Ig class (pan-Ig ECL) and the second multiplexing and discriminating Ig of different classes (IgM, IgA and IgG multiplexed-ECL). In both 2018 and 2020, pan-Ig ECL assays showed a relatively good performance, although lower than that of the best RBAs. Multiplexed IgG, IgM and IgA ECL assays instead were not only less sensitive, as might have been expected, but also less specific, as signalled by the scoring as IgM and/or IgA IAA positive of some of the included dilutions of HUI-018, a mouse anti-insulin mAb of the IgG1 class.

The remaining non-radioactive assays submitted to the workshops comprised a variety of formats such as CLIA, FloCMIA, LBI, ELISAs, and ELISAs using oxidised insulin as antigen. All of these assays showed a poor performance, with drastically reduced sensitivity and specificity compared with RBA and their ROC-AUCs demonstrated an inability to discriminate case sera from control sera.

In both workshops, we observed discrepancies of positive/negative scores and ranking of antibody levels across assays and formats, even when the analysis was limited to assays with overall good performance. The underlying reasons for the selective recognition of some case and control samples as IAA positive in certain assay formats but not others remain to be fully clarified. However, two main mechanisms can be put forward to explain these discrepancies: the first is simply linked to the difficulty of lower performance assays in identifying as positive sera presenting with low level IAA; and the second presumes a differential recognition of insulin epitopes by antibodies present only in a subset of sera. Multiple causes might underly this potential second mechanism, such as possible alteration of some insulin epitopes in certain assay formats caused by the addition of tags (e.g. biotin residues in ECL or luciferase enzyme in LIPS) or by potential alternative antigen post-translational modification(s) in the different expression systems used for their production. This potential format-associated selective recognition of some epitopes remains to be evaluated but might have an important impact on autoantibody-based screening strategies, which currently are still based on RBA.

In both the IASP 2018 and IASP 2020 workshops, the comparison of quantitative IAA results was complicated by the variety of local non-standardised arbitrary units and calculation algorithms into which results were expressed. In the absence of a WHO-recognised IAA standard serum, we explored the possibility of using an anti-insulin mouse monoclonal antibody as reference. For this reason, we distributed anonymised serial dilutions of the HUI-018 mAb. Most liquid-phase assays showed a clear ability to detect HUI-018 binding to insulin but the correct ranking of the mAb dilutions was challenging for assays with lower performance. While neither IASP study was designed to determine rigorously the analytical sensitivity of participating assays, using the differential recognition of the HUI-018 mAb dilution as a proxy, we could infer the presence of important differences in analytical sensitivity across IAA assays.

Moreover, the conversion of local units into common HUI-018 units further confirmed the lack of good quantitative concordance of IAA assays even when using the same format, a phenomenon that could not be conclusively clarified within the limits of the current study design.

Legislative and logistic pressure against the use of radioactive substances spur the development and validation of novel non-radioactive immunoassays. Furthermore, the expected future implementation of antibody-based population screening programmes for type 1 diabetes would benefit from the implementation of high-performance IAA assays dispensing with the need for radio-isotopic tracers. In this context, while in-house micro-assay RBAs still constituted the majority of the best-performing assays in 2020, some non-radioactive formats could indeed achieve both high sensitivity and specificity (e.g. ADAP, LIPS and ECL). However, none of the classical immunoassay formats widely adopted in routine clinical diagnostics (e.g. ELISA, CLIA and Luminex bead-based assays) nor assays aimed at measuring Ig class-specific autoantibody responses can be currently recommended for IAA measurement in light of their poor demonstrated performance.

In conclusion, our research supports the value of type 1 diabetes autoantibody assay evaluation programmes. These programmes not only help to assess the accuracy of diagnostic tests objectively but also provide academic research laboratories and companies with the chance to learn and improve their immunoassays.

## Supplementary information

ESM 1 (PDF 9.18 kb)

ESM 2 (XLSX 28 kb)

ESM 3 (XLSX 24 kb)

## Data Availability

The datasets generated and analysed during the current study are available from the corresponding author on reasonable request and provided the permission of all participating laboratories is given.

## Abbreviations

AC1: Gwet’s coefficient of inter-rater agreement reliability
ADAP: Antibody-dependent agglutination PCR
APPA: Average pairwise per cent agreement
AS95: Adjusted sensitivity at 95% specificity
AS99: Adjusted sensitivity at 99% specificity
AS100: Adjusted sensitivity at 100% specificity
CLIA: Chemiluminescence immunoassay
DASP: Diabetes Autoantibody Standardization Program
DK units: National Institute of Diabetes and Digestive and Kidney Diseases consortium units
ECL: Electrochemiluminescence
FDR: First-degree relative
FloCMIA: Flow cytometry microsphere immunoassay
IAS: Insulin autoimmune syndrome
IASP: Islet Autoantibody Standardization Program
IDS: Immunology of Diabetes Society
*k*: Fleiss’ coefficient of inter-rater agreement reliability
LBI: Luminex bead immunoassay
LCSP: Lowest Concentration Scored Positive
LIPS: Luciferase immunoprecipitation system
mAb: Monoclonal antibody
OCCC: Overall concordance correlation coefficient
pAUC95: Partial ROC-AUC at 95% specificity
RBA: Radiobinding assay
ROC-AUC: Area under the receiver operating characteristic curve
W: Kendall’s ranking agreement coefficient

## References

[1] Moinat P, Marston E (1958). A quantitative estimation of antibodies to exogenous insulin in diabetic subjects. Diabetes.

[2] Cappellani D, Macchia E, Falorni A, Marchetti P (2020). Insulin autoimmune syndrome (Hirata disease): a comprehensive review fifty years after its first description. Diabetes Metab Syndr Obes Targets Ther.

[3] Palmer JP, Asplin CM, Clemons P (1983). Insulin antibodies in insulin-dependent diabetics before insulin treatment. Science.

[4] Censi S, Mian C, Betterle C (2018). Insulin autoimmune syndrome: from diagnosis to clinical management. Ann Transl Med.

[5] Vehik K, Bonifacio E, Lernmark Å (2020). Hierarchical order of distinct autoantibody spreading and progression to type 1 diabetes in the TEDDY study. Diabetes Care.

[6] Krischer JP, Liu X, Lernmark Å (2021). Characteristics of children diagnosed with type 1 diabetes before vs after 6 years of age in the TEDDY cohort study. Diabetologia.

[7] Achenbach P, Koczwara K, Knopff A, Naserke H (2004). Mature high-affinity immune responses to (pro) insulin anticipate the autoimmune cascade that leads to type 1 diabetes. J Clin Invest.

[8] Schlosser M, Koczwara K, Kenk H (2005). In insulin-autoantibody-positive children from the general population, antibody affinity identifies those at high and low risk. Diabetologia.

[9] Achenbach P, Schlosser M, Williams AJK (2007). Combined testing of antibody titer and affinity improves insulin autoantibody measurement: Diabetes Antibody Standardization Program. Clin Immunol.

[10] Elding Larsson H, Vehik K, Gesualdo P (2014). Children followed in the TEDDY study are diagnosed with type 1 diabetes at an early stage of disease: Intense follow-up enables early detection of type 1 diabetes. Pediatr Diabetes.

[11] Krischer JP, Liu X, Vehik K (2019). Predicting islet cell autoimmunity and type 1 diabetes: an 8-year TEDDY study progress report. Diabetes Care.

[12] Ziegler A-G, Kick K, Bonifacio E (2020). Yield of a public health screening of children for islet autoantibodies in Bavaria, Germany. JAMA.

[13] Karpen SR, Dunne JL, Frohnert BI et al (2022) Consortium-based approach to receiving an EMA qualification opinion on the use of islet autoantibodies as enrichment biomarkers in type 1 diabetes clinical studies. Diabetologia. 10.1007/s00125-022-05751-0 10.1007/s00125-022-05751-0 PMC10024532 35867129

[14] Wilkin T, Palmer J, Bonifacio E (1987). First international workshop on the standardisation of insulin autoantibodies. Diabetologia.

[15] Wilkin T, Palmer J, Kurtz A, Bonifacio E, Diaz JL (1988). The second international workshop on the standardisation of insulin autoantibody (IAA) measurement. Held in New York, 27-30 October 1987. Diabetologia.

[16] Greenbaum CJ, Palmer JP, Kuglin B, Kolb H (1992). Insulin autoantibodies measured by radioimmunoassay methodology are more related to insulin-dependent diabetes mellitus than those measured by enzyme-linked immunosorbent assay: results of the Fourth International Workshop on the Standardization of Insulin. J Clin Endocrinol Metab.

[17] Greenbaum CJ, Wilkin TJ, Palmer JP (1992). Fifth international serum exchange workshop for insulin autoantibody (IAA) standardization. The immunology and diabetes workshops and participating laboratories. Diabetologia.

[18] Wilkin TJ, Schoenfeld SL, Diaz JL, Kruse V, Bonifacio E, Palmer JP (1989). Systematic variation and differences in insulin-autoantibody measurements. Diabetes.

[19] Bingley PJ, Bonifacio E, Mueller PW (2003). Diabetes antibody standardization program: first assay proficiency evaluation. Diabetes.

[20] Schlosser M, Mueller PW, Törn C, Bonifacio E, Bingley PJ, and participating laboratories (2010). Diabetes antibody standardization program: evaluation of assays for insulin autoantibodies. Diabetologia.

[21] Lampasona V, Pittman DL, Williams AJ (2019). Islet autoantibody standardization program 2018 workshop: interlaboratory comparison of glutamic acid decarboxylase autoantibody assay performance. Clin Chem.

[22] World Medical Association (2001) World Medical Association declaration of Helsinki. Ethical principles for medical research involving human subjects. Bull World Health Organ 79(4):373–374 PMC2566407 11357217

[23] Johansson E, Wu X, Yu B (2021). Insulin binding to the analytical antibody sandwich pair OXI-005 and HUI-018: Epitope mapping and binding properties. Protein Sci.

[24] Bonifacio E, Yu L, Williams AK (2010). Harmonization of glutamic acid decarboxylase and islet antigen-2 autoantibody assays for national institute of diabetes and digestive and kidney diseases consortia. J Clin Endocrinol Metab.

[25] R Core Team (2020). R: A Language and Environment for Statistical Computing.

[26] Wongpakaran N, Wongpakaran T, Wedding D, Gwet KL (2013). A comparison of Cohen’s Kappa and Gwet’s AC1 when calculating inter-rater reliability coefficients: a study conducted with personality disorder samples. BMC Med Res Methodol.

[27] Cicchetti DV, Feinstein AR (1990). High agreement but low kappa: II. Resolving the paradoxes. J Clin Epidemiol.

[28] Gamer M, Lemon J, Fellows I, Singh P (2019) irr: Various Coefficients of Interrater Reliability and Agreement. R package version 0.84.1. Available from: https://CRAN.R-project.org/package=irr (accessed on 26 January 2019)

[29] Ma H, Bandos AI, Rockette HE, Gur D (2013). On use of partial area under the ROC curve for evaluation of diagnostic performance. Stat Med.

[30] Robin X, Turck N, Hainard A (2011). pROC: an open-source package for R and S+ to analyze and compare ROC curves. BMC Bioinformatics.

[31] Kendall MG, Babington Smith B (1939). The problem of m rankings. Ann Math Stat.

[32] Stevenson M, Sergeant E, Nunes T et al (2021) epiR: tools for the analysis of epidemiological data. R package version 2.0.26. https://CRAN.R-project.org/package=epiR (accessed on 15 July 2021)

[33] Williams AJ, Bingley PJ, Bonifacio E, Palmer JP, Gale EA (1997). A novel micro-assay for insulin autoantibodies. J Autoimmun.

[34] Tsai C, Robinson PV, Spencer CA, Bertozzi CR (2016). Ultrasensitive antibody detection by agglutination-PCR (ADAP). ACS Cent Sci.

[35] Liberati D, Wyatt RC, Brigatti C (2018). A novel LIPS assay for insulin autoantibodies. Acta Diabetol.

[36] Yu L, Dong F, Miao D, Fouts AR, Wenzlau JM, Steck AK (2013). Proinsulin/insulin autoantibodies measured with electrochemiluminescent assay are the earliest indicator of prediabetic islet autoimmunity. Diabetes Care.

[37] Strollo R, Vinci C, Napoli N, Pozzilli P, Ludvigsson J, Nissim A (2017). Antibodies to post-translationally modified insulin as a novel biomarker for prediction of type 1 diabetes in children. Diabetologia.

[38] Sabljic AV, Bombicino SS, Marfía JI (2021). Novel flow cytometric immunoassay for detection of proinsulin autoantibodies in diabetes mellitus employing a recombinant autoantigen expressed in E. coli. Front Immunol.

[39] TEDDY Study Group (2008). The environmental determinants of diabetes in the young (TEDDY) study. Ann N Y Acad Sci.

[40] Skyler JS, Greenbaum CJ, Lachin JM (2008). Type 1 Diabetes TrialNet--an international collaborative clinical trials network. Ann N Y Acad Sci.

