# Supplementary material for: Islet Autoantibody Standardization Program: interlaboratory comparison of insulin autoantibody assay performance in 2018 and 2020 workshops
Source: Diabetologia. 2023 Feb 10;66(5):897–912. doi: 10.1007/s00125-023-05877-9 (PMC10036445; doi:10.1007/s00125-023-05877-9)
Supplement: Supplementary file 1 — (PDF 9.18 kb) [file 125_2023_5877_MOESM1_ESM.pdf]

New onset T1D cases	Multiple auto-Ab$^+$ First Degree relatives	Blood donor controls
IDS004	IDS316	LQ22722
IDS005	IDS317	
IDS006	IDS320	
IDS007	IDS324	
IDS009	IDS325	
IDS285	IDS327	
IDS295		
IDS299		
IDS302		
IDS303		
IDS304		
IDS309		
IDS310		
IDS311		
IDS312		
IDS314		
IDS318		
IDS322		
IDS326		
IDS328		

ESM table 1: ID of samples included in both IASP2018 and IASP2020 sample sets (21 new onset T1D cases, 6 multiple auto-Ab$^+$ FDR, 1 blood donor control)

ESM Table 4: assay agreement and concordance in new onset T1D and multiple auto-Ab^{+} cases stratified by format in IASP2018

Format	n. assays	APPA[a]	AC1[b]	*k*[c]	W[d]	OCCC[e]
Radio Binding Assay (RBA)						
Local	13	74.5	0.52	0.469	0.694	0.319
Commercial	2	91.8	0.88	0.728	0.589	-
Antibody Dependent Agglutination PCR (ADAP)	1	-	-	-	-	-
Luciferase Immuno Precipitation System (LIPS)						
Insulin B NLuc reporter	2	66.0	0.33	0.313	0.918	0.103
Insulin A NLuc reporter	2	80.0	0.60	0.597	0.948	0.497
Proinsulin B NLuc reporter	5	88.8	0.78	0.776	0.943	0.398
Electro Chemi Luminescence (ECL)						
ECL	4[f]	62.6	0.29	0.208	0.642	0.107
ECL IgG specific	2[f]	90.0	0.82	0.774	0.916	-
ECL IgM specific	2[f]	94.0	0.93	0.694	0.813	-
ECL IgA specific	2[f]	94.0	0.94	-0.031	0.670	-
Chemi Luminescence Immuno Assay (CLIA)	2	78.0	0.73	-0.124	0.562	-
ELISA						
Insulin	2	92.0	0.87	0.802	0.952	-
HOCl-insulin	2	86.0	0.76	0.673	0.958	-
OH-insulin	2	90.0	0.86	0.675	0.958	-
Luminex Bead Immunoassay (LBI)	2	98.0	0.98	0.878	0.994	-
All assays median (IQR)	45	89.4 (78.5 –92.0)	0.80 (0.63 - 0.88)	0.674 (0.352-0.763)	0.917 (0.676-0.951)	0.319 (0.107-0.398)

[a]APPA: Average Pairwise Percent Agreement (%)
[b]AC1: Gwet's agreement coefficient
[c]*k*: Fleiss *k* agreement coefficient
[d]W: Kendall's W rank agreement coefficient (in assays with reported units)
[e]OCCC: Barnhart's Overall Concordance correlation Coefficient (in the portion of assays selected as described)
[f] pan-Ig and Ig-specific ECL assays using either insulin or proinsulin were conflated

ESM Table 5: assay agreement and concordance in healthy controls stratified by format in IASP2018

Format	n. assays	APPA[a]	AC1[b]	*k*[c]	W[d]	OCCC[e]
Radio Binding Assay (RBA)						
Local	13	95.5	0.95	0.139	0.136	0.031
Commercial	2	100	NA	NA	0.246	-
Antibody Dependent Agglutination PCR (ADAP)	1	-	-	-	-	-
Luciferase Immuno Precipitation System (LIPS)						
Insulin B NLuc reporter	2	93.3	0.93	-0.035	0.482	0.087
Insulin A NLuc reporter	2	97.8	0.98	0.489	0.432	0.282
Proinsulin B NLuc reporter	5	97.8	0.98	0.694	0.438	0.080
Electro Chemi Luminescence (ECL)						
ECL	4[f]	77.6	0.71	-0.024	0.282	-0.003
ECL IgG specific	2[f]	96.7	0.96	0.782	0.809	-
ECL IgM specific	2[f]	92.2	0.91	0.181	0.831	-
ECL IgA specific	2[f]	76.7	0.71	-0.132	0.701	-
Chemi Luminescence Immuno Assay (CLIA)	2	47.8	0.05	-0.165	0.569	-
ELISA						
Insulin	2	92.2	0.90	0.675	0.949	-
HOCl-insulin	2	92.2	0.89	0.727	0.962	-
OH-insulin	2	97.8	0.97	0.888	0.925	-
Luminex Bead Immunoassay (LBI)	2	95.6	0.94	0.793	0.995	-
All assays – median (IQR)	45	94.4 (92.2 – 97.5)	0.93 (0.89 - 0.96)	0.489 (-0.024-0.727)	0.635 (0.434-0.902)	0.080 (0.031 – 0.087)

[a]APPA: Average Pairwise Percent Agreement (%)
[b]AC1: Gwet's agreement coefficient
[c]*k*: Fleiss *k* agreement coefficient
[d]W: Kendall's W rank agreement coefficient (in assays with reported units)
[e]OCCC: Barnhart's Overall Concordance correlation Coefficient (in the portion of assays selected as described)
[f] pan-Ig and Ig-specific ECL assays using either insulin or proinsulin were conflated

ESM Table 6: assay agreement and concordance in new onset T1D and multiple auto-Ab$^+$ cases stratified by format in IASP2020

Format	n. assays	APPA[a]	AC1[b]	*k*[c]	W[d]	OCCC[e]
Radio Binding Assay (RBA)						
Local	10	74.1	0.48	0.481	0.708	0.376
Commercial	3	97.3	0.96	0.893	0.498	-
Antibody Dependent Agglutination PCR (ADAP)	1	-	-	-	-	-
Luciferase Immuno Precipitation System (LIPS)						
Insulin B NLuc reporter	3	84.0	0.68	0.675	0.860	0.737
Insulin B NLuc reporter (multiplexed)	2	92.0	0.84	0.836	0.908	0.847
Proinsulin B NLuc reporter	6	85.3	0.71	0.701	0.851	0.201
Electro Chemi Luminescence (ECL)						
ECL	4[f]	64.3	0.30	0.270	0.448	-0.004
ECL IgG specific	2[f]	76.0	0.54	0.491	0.845	-
ECL IgM specific	2[f]	70.0	0.57	0.025	0.532	-
ECL IgA specific	2[f]	90.0	0.89	-0.053	0.509	-
Chemi Luminescence Immuno Assay (CLIA)	1	-	-	-	-	-
Flow Cytometry Microsphere Immuno Assay (FloCMIA)	1	-	-	-	-	-
All assays median (IQR)	37	84.0 (74.1 – 90.0)	0.68 (0.54 - 0.84)	0.491 (0.270-0.701)	0.708 (0.509 – 0.851)	0.376 (0.201 – 0.737)

[a]APPA: Average Pairwise Percent Agreement (%)
[b]AC1: Gwet's agreement coefficient
[c]*k:* Fleiss *k* agreement coefficient
[d]W: Kendall's W rank agreement coefficient
[e]OCCC: Barnhart's Overall Concordance correlation Coefficient (in a portion of assays selected as described)
[f]pan-Ig and Ig-specific ECL assays using either insulin or proinsulin were conflated

ESM Table 7: assay agreement and concordance in healthy controls stratified by format in IASP2020

Format	# assays	APPA[a]	AC1[b]	*k* [c]	W[d]	OCCC[e]
Radio Binding Assay (RBA)						
Local	10	98.7	0.99	-0.007	0.150	0.051
Commercial	3	100	NA	NA	0.107	-
Antibody Dependent Agglutination PCR (ADAP)	1	-	-	-	-	-
Luciferase Immuno Precipitation System (LIPS)						
Insulin B NLuc reporter	3	94.1	0.94	0.169	0.478	0.268
Insulin B NLuc reporter (multiplexed)	2	96.7	0.97	-0.017	0.265	0.359
Proinsulin B NLuc reporter	6	96.3	0.96	0.091	0.332	0.030
Electro Chemi Luminescence (ECL)						
ECL	4[f]	92.0	0.91	0.003	0.182	-0.002
ECL IgG specific	2[f]	93.3	0.92	0.535	0.203	-
ECL IgM specific	2[f]	85.6	0.83	-0.078	0.378	-
ECL IgA specific	2[f]	95.6	0.95	-0.023	0.401	-
Chemi Luminescence Immuno Assay (CLIA)	1	-	-	-	-	-
Flow Cytometry Microsphere Immuno Assay (FloCMIA)	1	-	-	-	-	-
All assays median (IQR)	37	95.6 (93.3 – 96.7)	0.95 (0.92- 0.96)	-0.002 (-0.019-0.111)	0.265 (0.182-0.378)	0.051 (0.030 – 0.268)

[a]APPA: Average Pairwise Percent Agreement (%)
[b]AC1: Gwet's agreement coefficient
[c]*k*: Fleiss *k* agreement coefficient
[d]W: Kendall's W rank agreement coefficient
[e]OCCC: Barnhart's Overall Concordance correlation Coefficient (in a portion of assays selected as described)
[f]pan-Ig and Ig-specific ECL assays using either insulin or proinsulin were conflated

ESM Figure 1

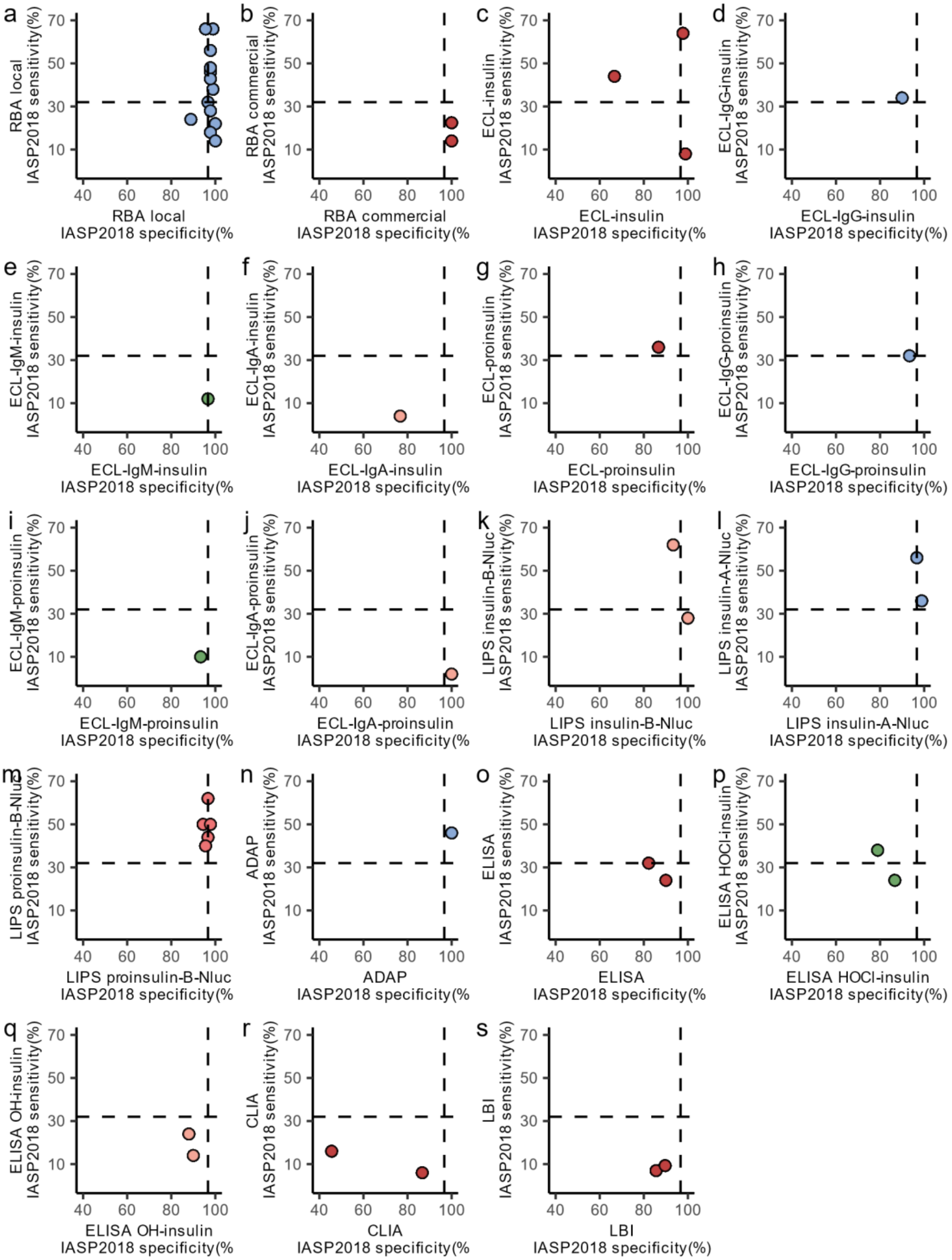

ESM Figure 1. Sensitivity and specificity of IAA assays submitted to IASP2018.
Assays are grouped in panels according to format and its variants: RBA (a-b), ECL (c-j), LIPS (k-m), ADAP (n), ELISA (o-q), CLIA (r), LBI (s). Circles show sensitivity and specificity of each submitted assay calculated from laboratory assigned positive/negative sample scores. Horizontal and vertical dashed lines correspond to the median sensitivity and specificity calculated across all assays in IASP2018.

ESM Figure 2

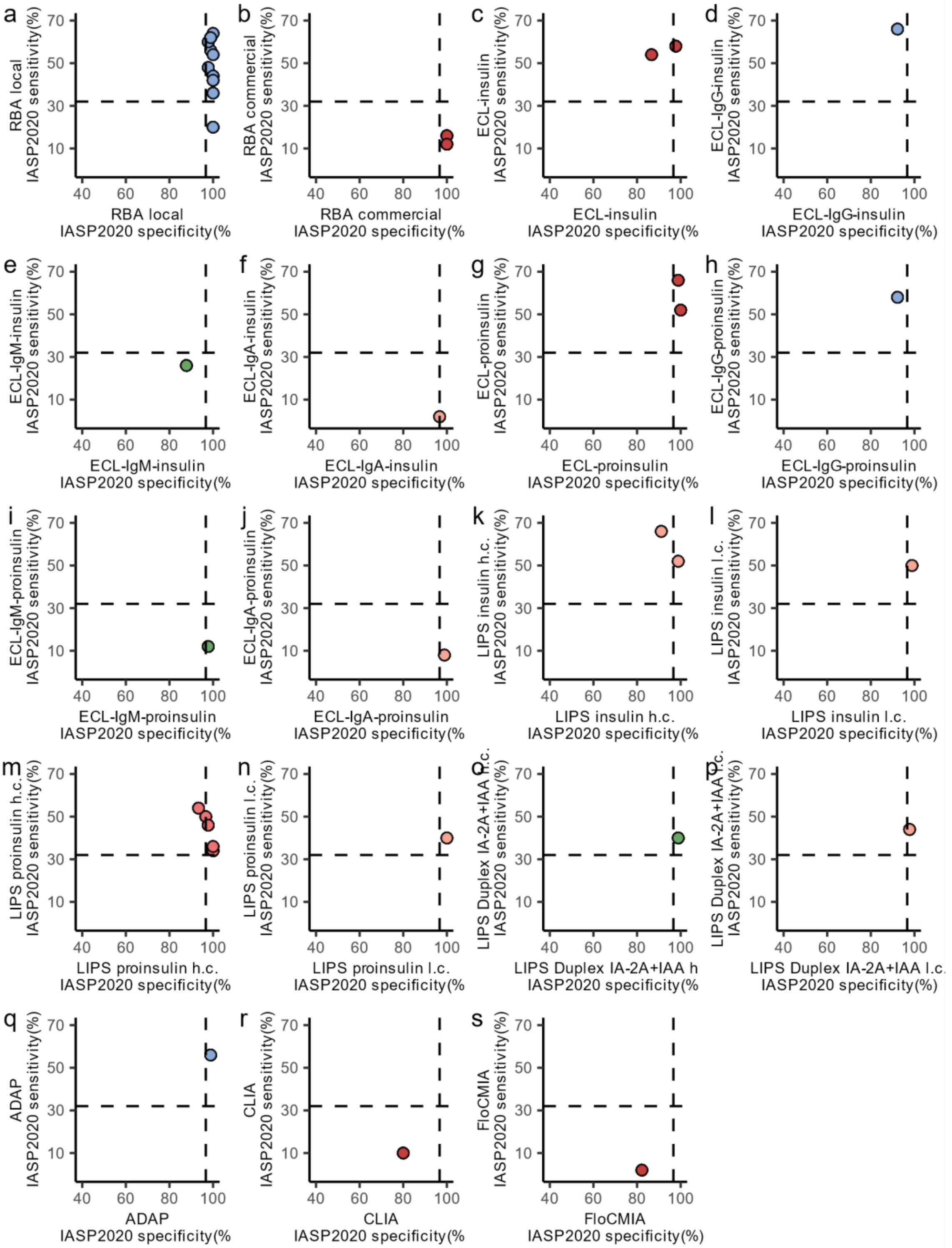

ESM Figure 2. Sensitivity and specificity of IAA assays submitted to IASP2020.
Assays are grouped in panels according to format and its variants: RBA (a-b), ECL (c-j), LIPS (k-p), ADAP (q), CLIA (r), FloCMIA (s). Circles show sensitivity and specificity of each submitted assay calculated from laboratory assigned positive/negative sample scores. Horizontal and vertical dashed lines correspond to the median sensitivity and specificity calculated across all assays in IASP2020.

ESM Figure 3

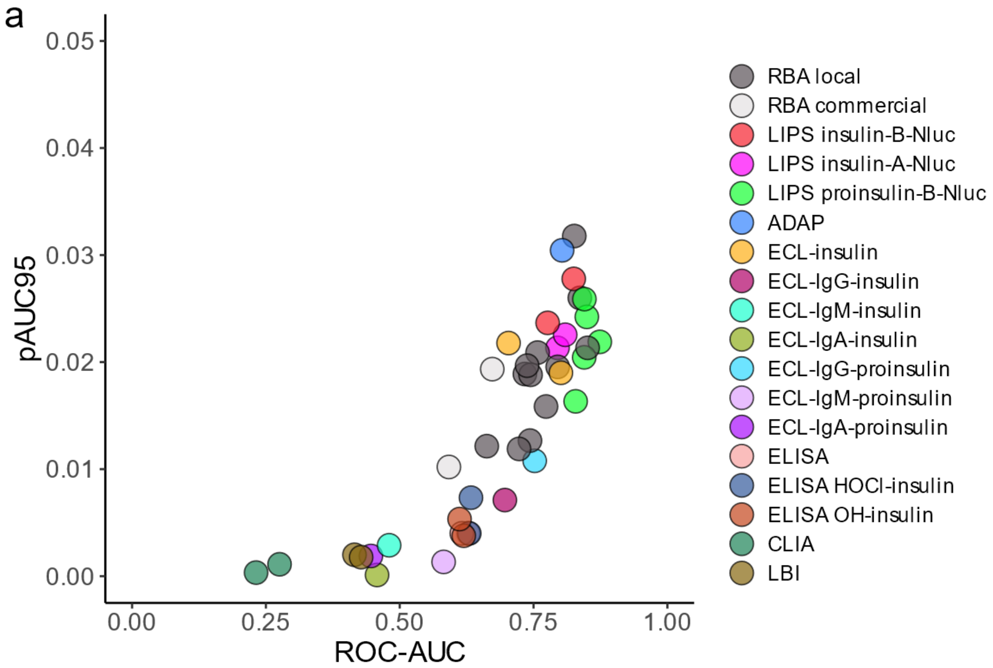

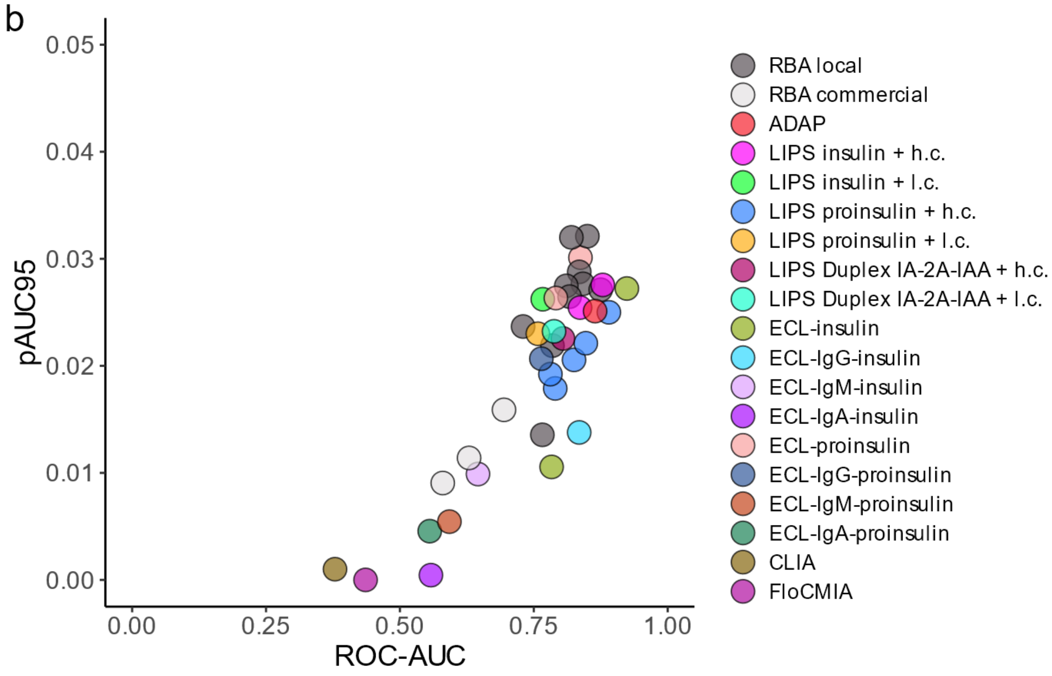

ESM Figure 3. ROC-AUC and pAUC95 of IAA assays submitted to IASP2018 (a) and IASP2020 (b).
Scatterplot of each submitted assay ROC-AUC and pAUC95. Circles show the results of each IAA assay. Circle fill is assigned according to assay format and its variants.

ESM Figure 4

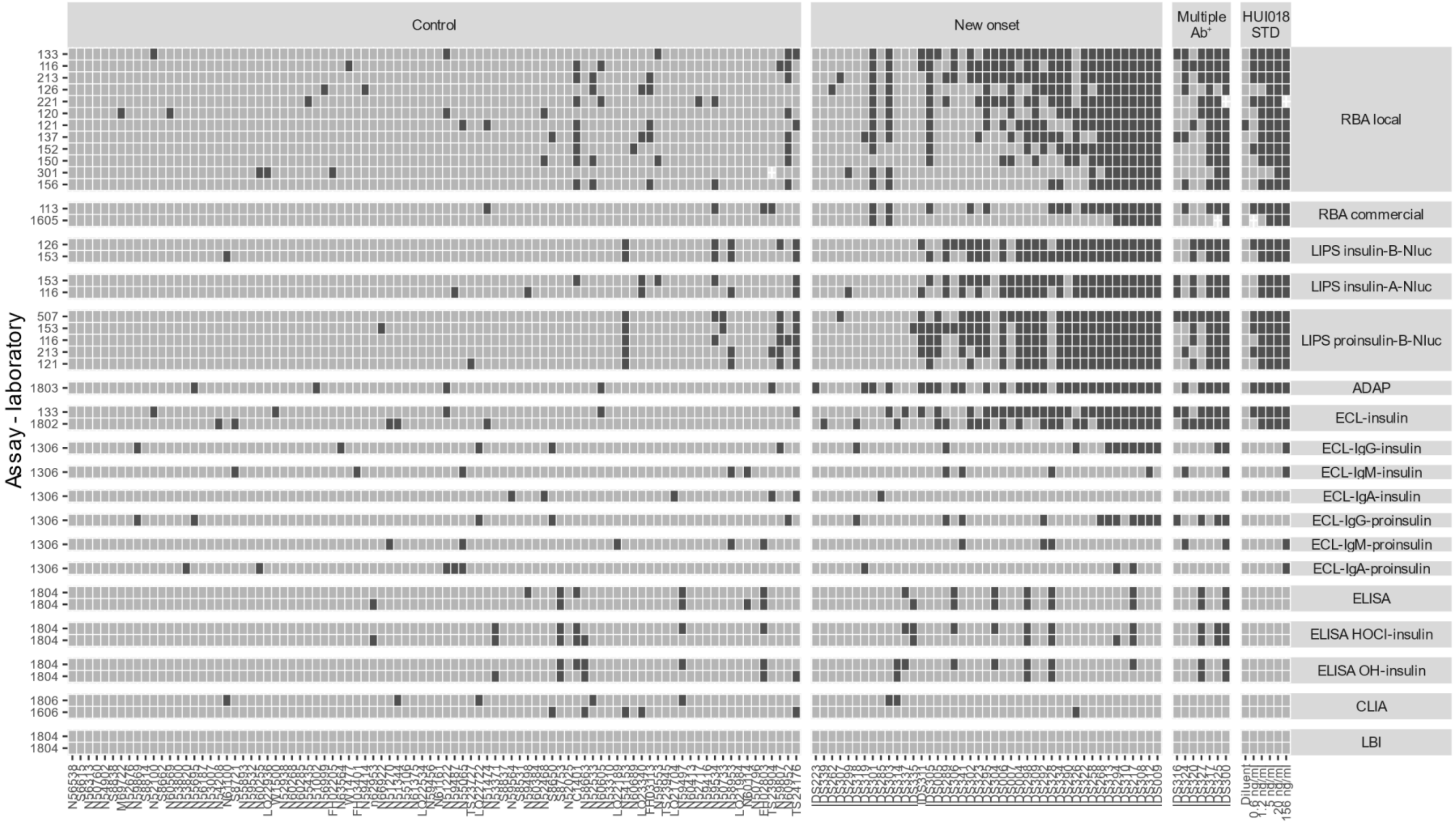

ESM figure 4. IASP2018 tilemap of IAA scores after imposing the threshold for positivity at 95% of specificity (AS95).
Tilemap of IAA positive (dark grey) or negative (light grey) scores assigned after placing the threshold for positivity at the 95th percentile of IAA units in blood donor samples. Samples in each indicated group (blood donor controls, new onset T1D, multiple autoantibody positive subjects, HUI-018 standards) are sorted on the horizontal axis according to their calculated median rank within each group. Assays are grouped by format and format variants and the groups are sorted on the vertical axis according to descending median performance (pAUC95).

ESM Figure 5

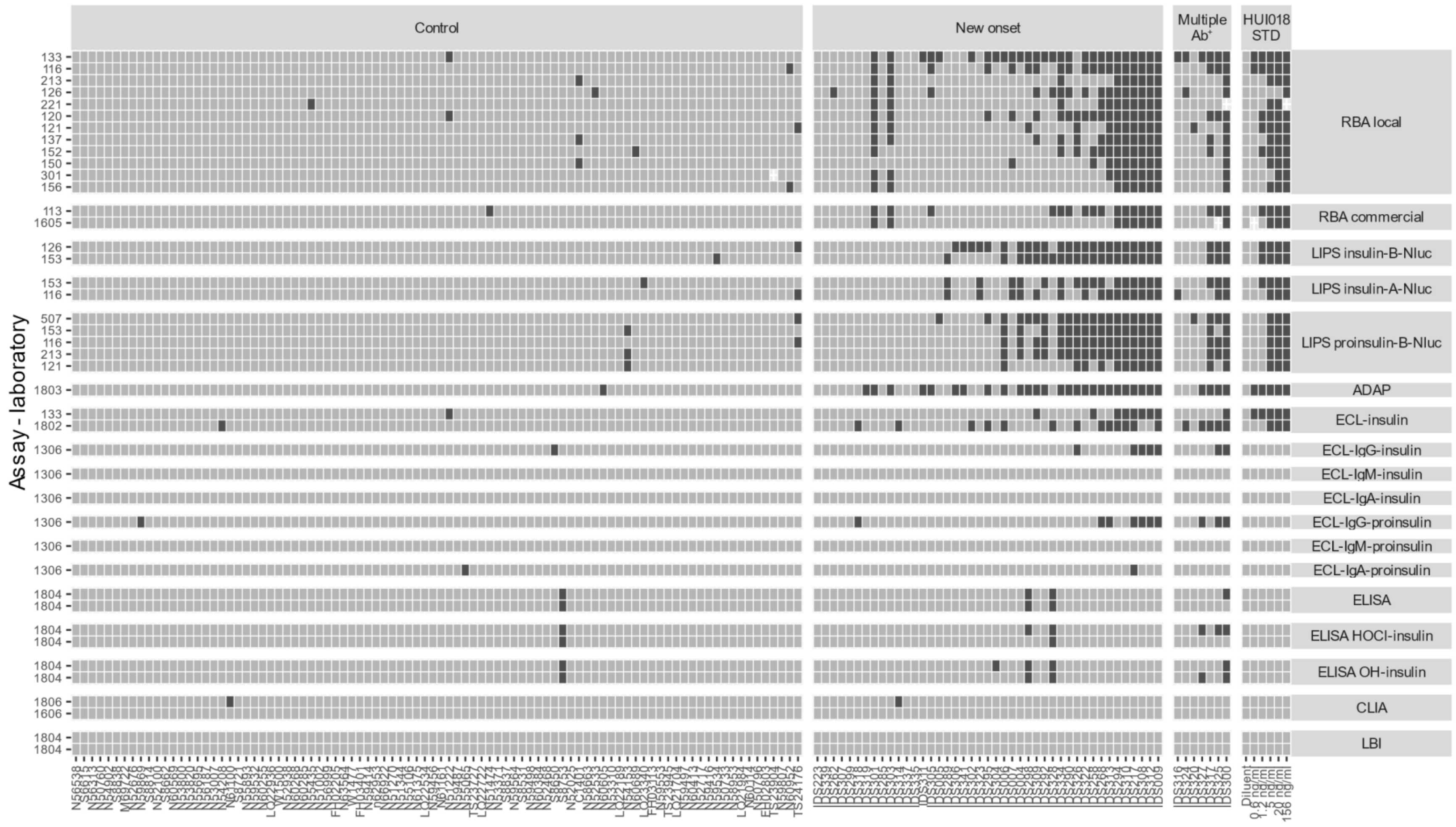

ESM figure 5. IASP2018 tilemap of IAA scores after imposing the threshold for positivity at 99% of specificity (AS99).
Tilemap of IAA positive (dark grey) or negative (light grey) scores assigned after placing the threshold for positivity at the 99th percentile of IAA units in blood donor samples. Samples in each indicated group (blood donor controls, new onset T1D, multiple autoantibody positive subjects, HUI-018 standards) are sorted on the horizontal axis according to their calculated median rank within each group. Assays are grouped by format and format variants and the groups are sorted on the vertical axis according to descending median performance (pAUC95).

ESM Figure 6

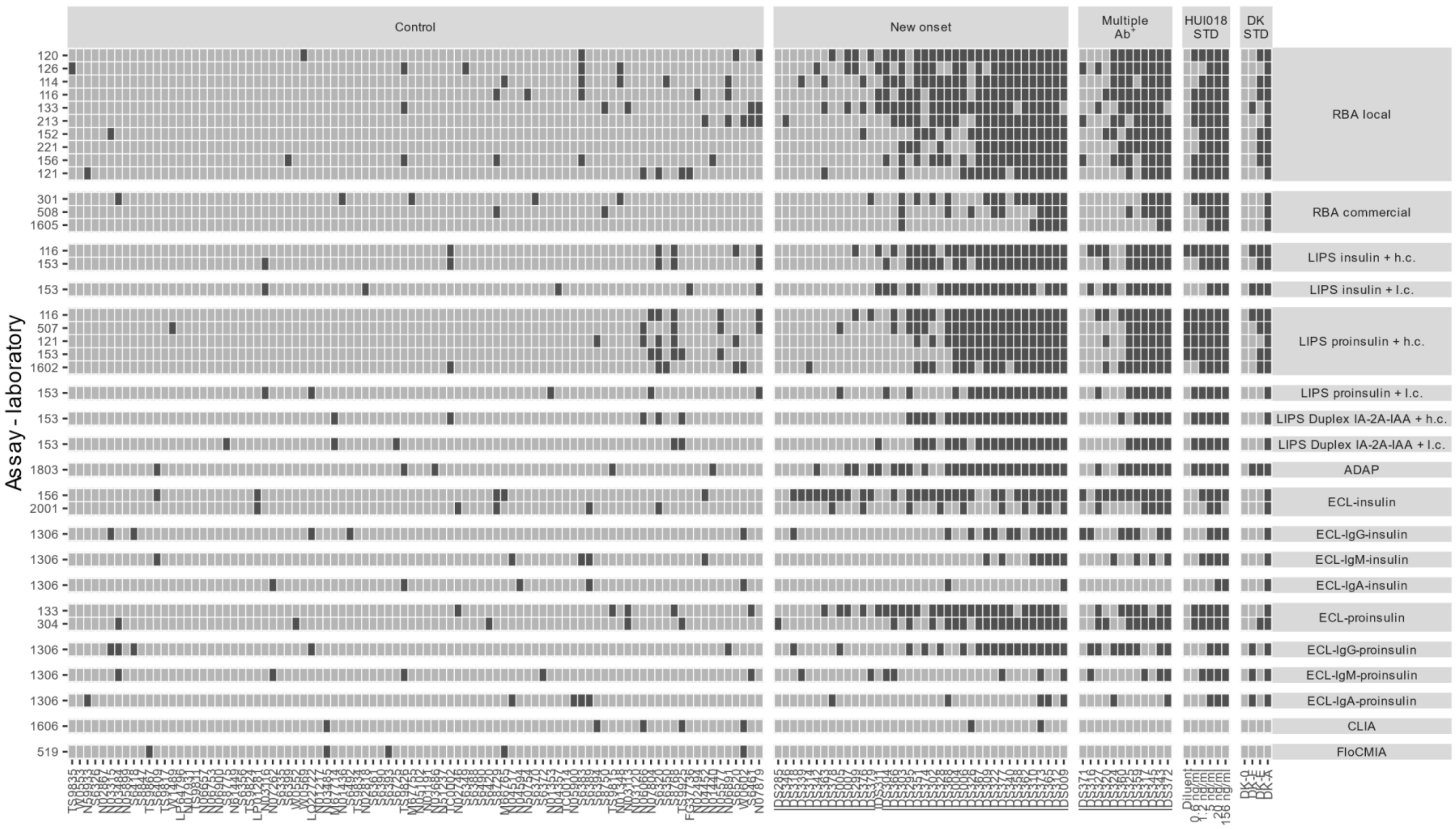

ESM figure 6. IASP2020 tilemap of IAA scores after imposing the threshold for positivity at 95% of specificity (AS95).
Tilemap of IAA positive (dark grey) or negative (light grey) scores assigned after placing the threshold for positivity at the 95th percentile of IAA units in blood donor samples. Samples in each indicated group (blood donor controls, new onset T1D, multiple autoantibody positive subjects, HUI-018 standards, DK standards) are sorted on the horizontal axis according to their calculated median rank within each group. Assays are grouped by format and format variants and the groups are sorted on the vertical axis according to descending median performance (pAUC95).

ESM Figure 7

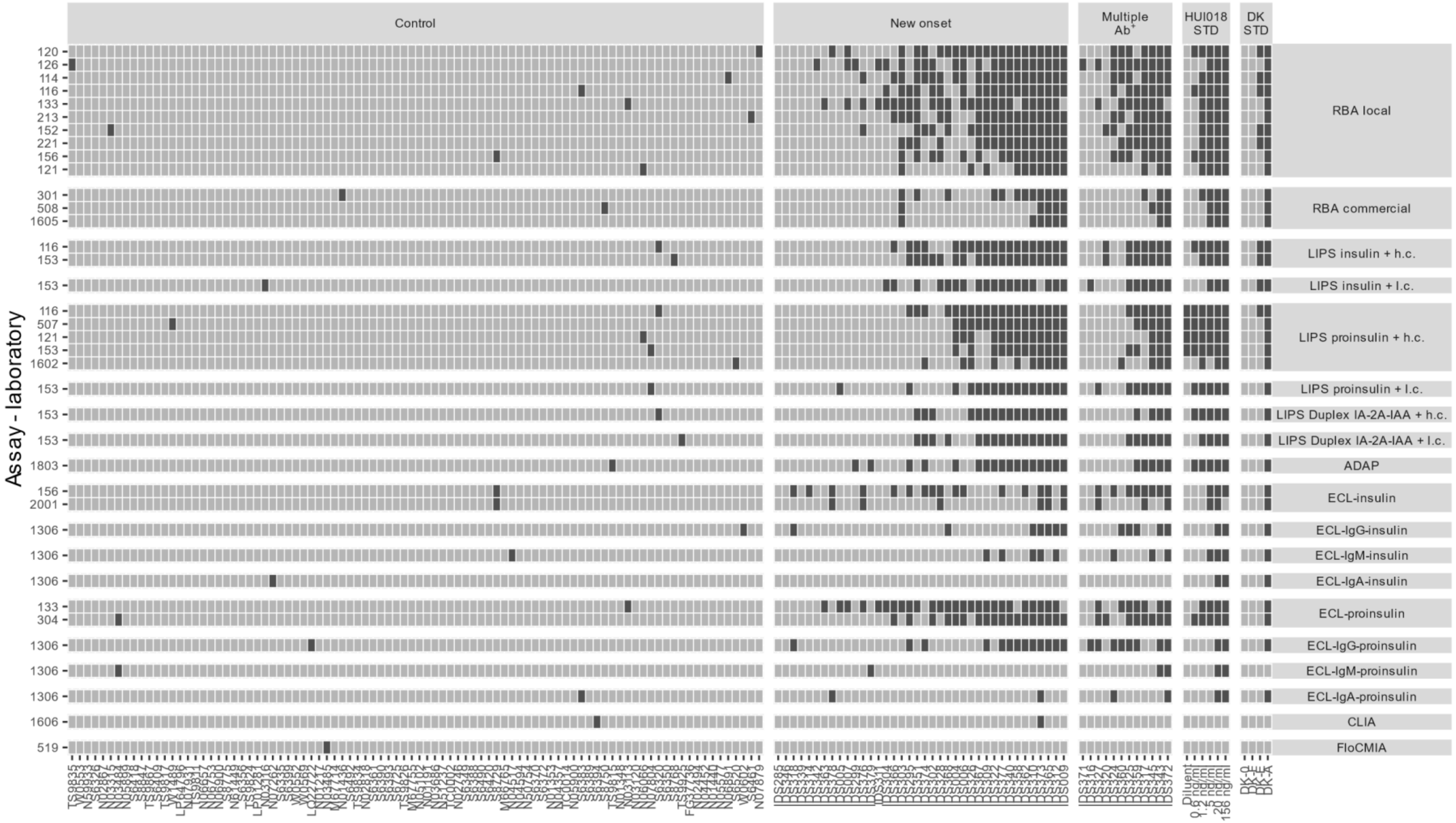

ESM figure 7. IASP2020 tilemap of IAA scores after imposing the threshold for positivity at 99% of specificity (AS99).
Tilemap of IAA positive (dark grey) or negative (light grey) scores assigned after placing the threshold for positivity at the 99th percentile of IAA units in blood donor samples. Samples in each indicated group (blood donor controls, new onset T1D, multiple autoantibody positive subjects, HUI-018 standards, DK standards) are sorted on the horizontal axis according to their calculated median rank within each group. Assays are grouped by format and format variants and the groups are sorted on the vertical axis according to descending median performance (pAUC95).

ESM Figure 8

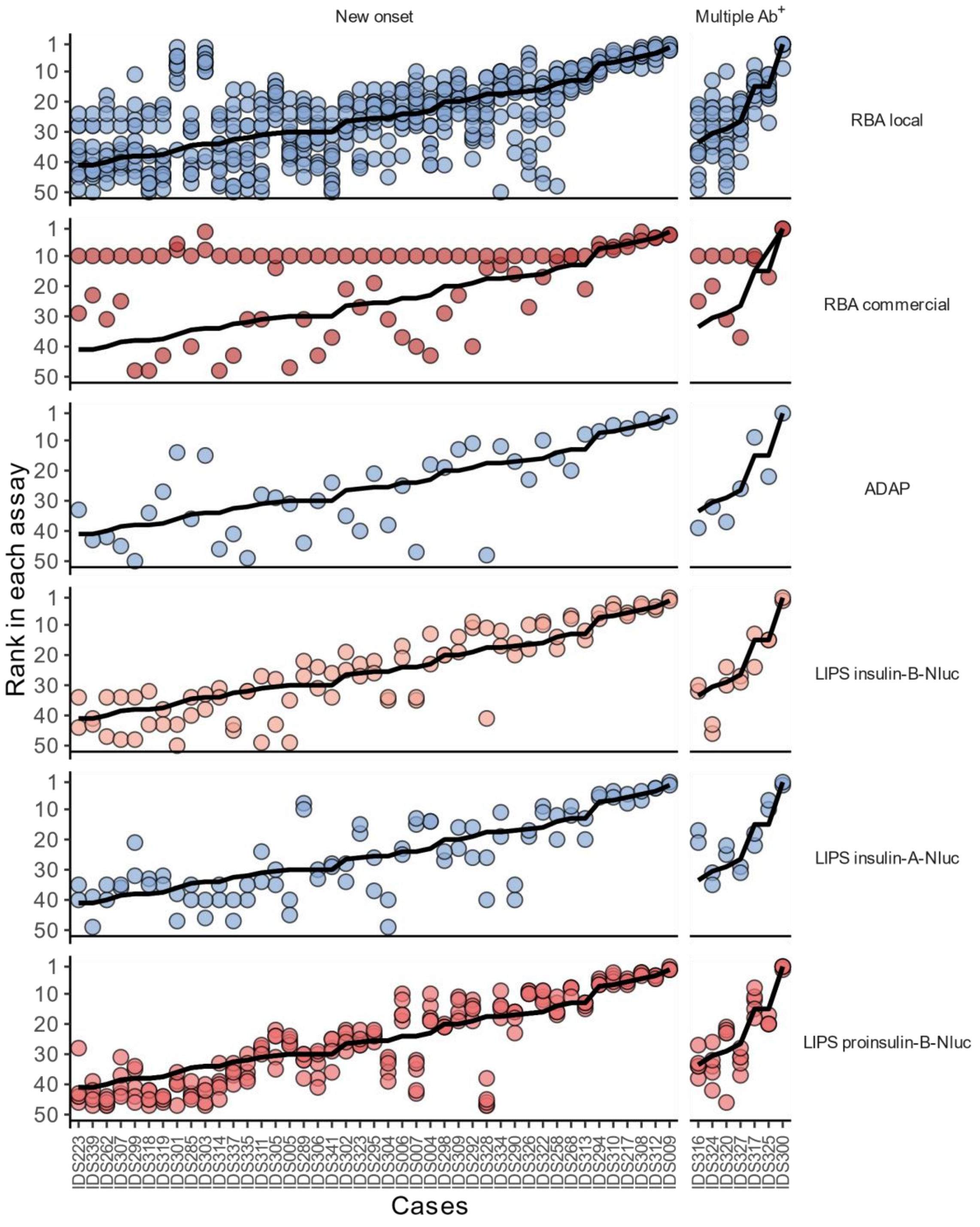

ESM figure 8. Rankings of IAA results in case samples included in IASP2018 (RBA, LIPS and ADAP assays).
Circles show the rank of sample in each assay belonging to the corresponding assay format or format variant group. Samples are grouped into new onset T1D and multiple autoantibody positive and within each group are sorted on the horizontal axis according to their median rank as calculated across all IASP2018 assays. The global median rank of cases in 2018 is shown as a solid black line.

ESM Figure 9

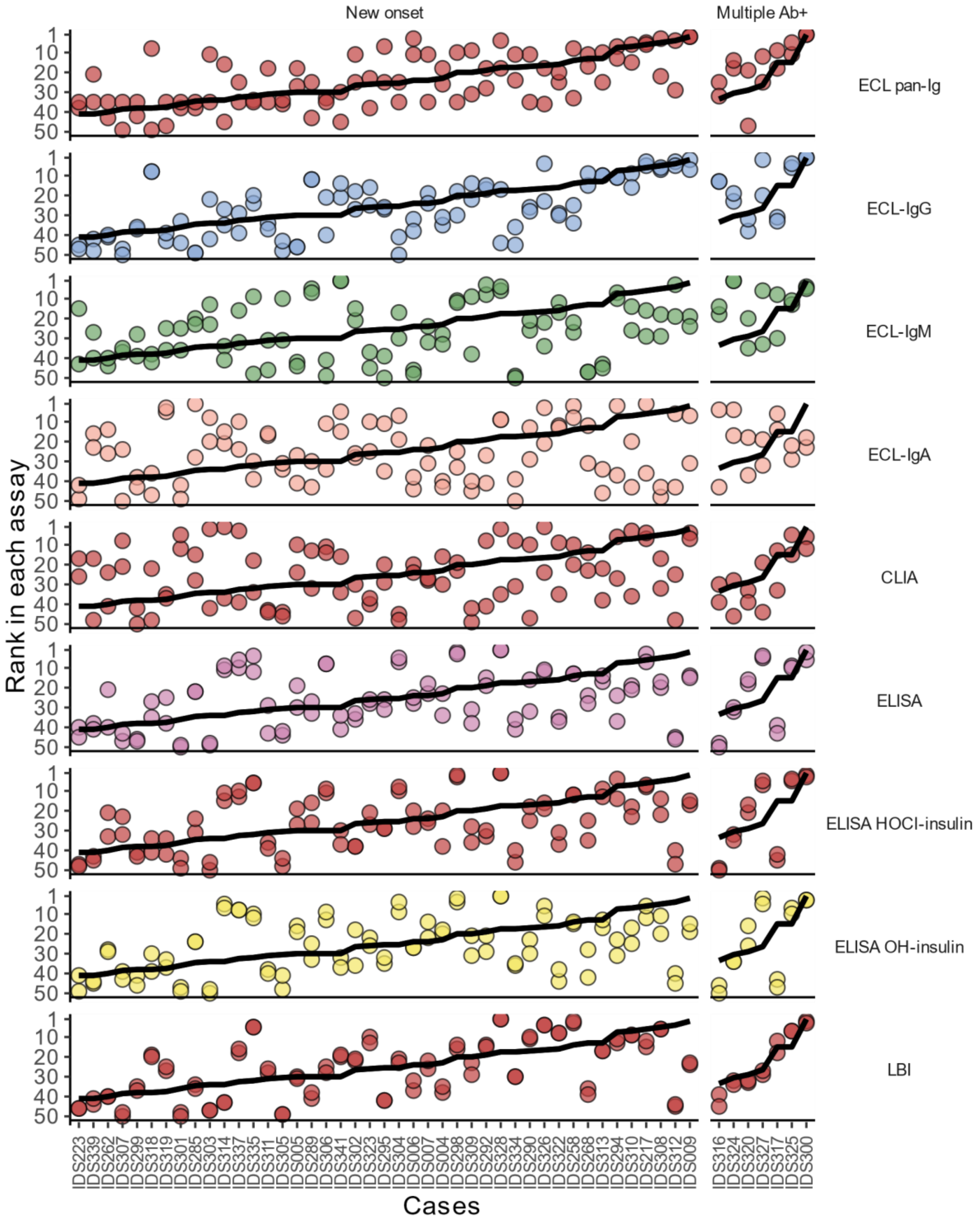

ESM figure 9. Rankings of IAA results in case samples included in IASP2018 (ECL, CLIA, ELISA and LBI assays).

Circles show the rank of sample in each assay belonging to the corresponding assay format or format variant group. Samples are grouped into new onset T1D and multiple autoantibody positive and within each group are sorted on the horizontal axis according to their median rank as calculated across all IASP2018 assays. The global median rank of cases in 2018 is shown as a solid black line.

ESM Figure 10

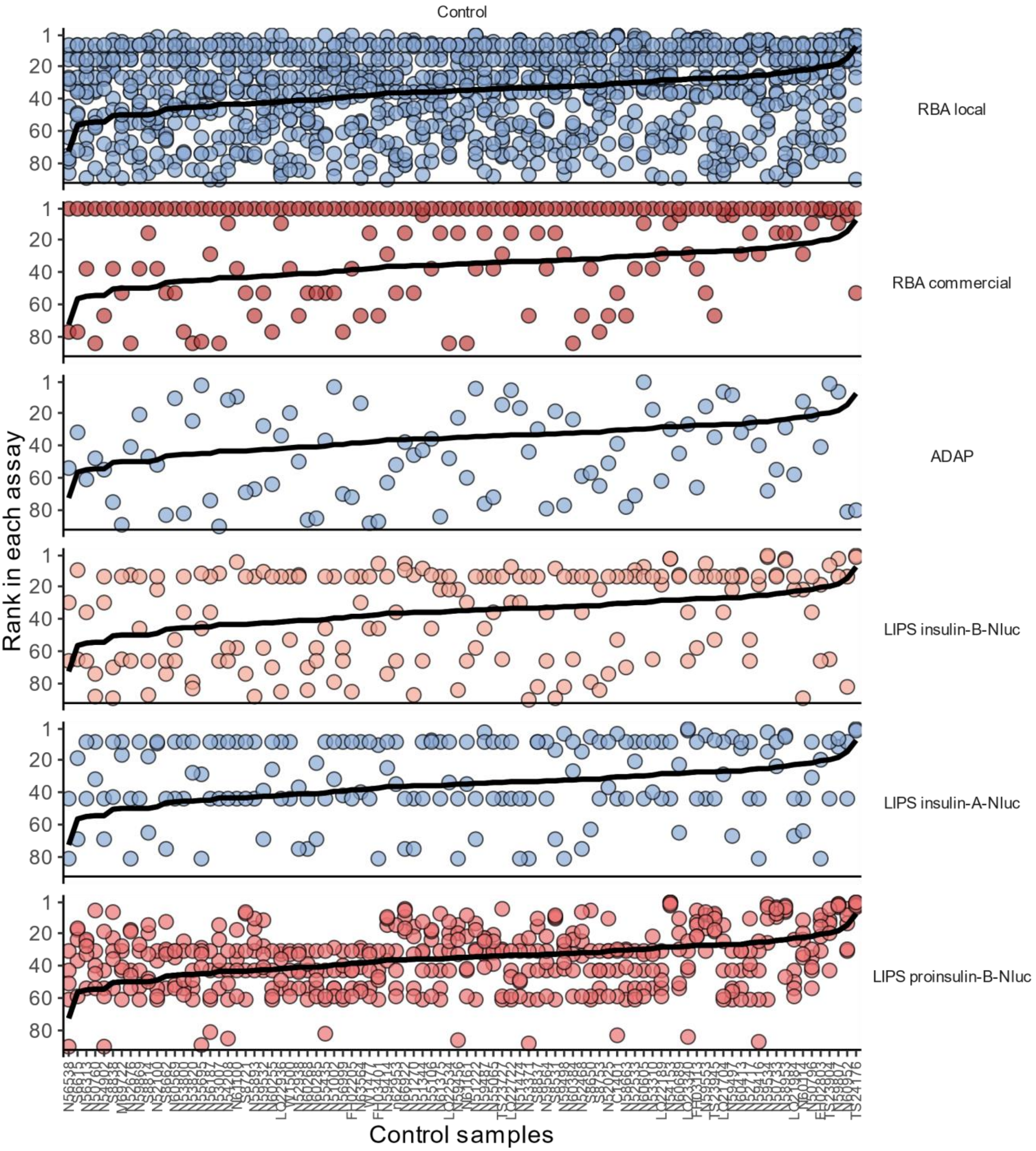

ESM figure 10. Rankings of IAA results in control samples included in IASP2018 (RBA, LIPS and ADAP assays).

Circles show the rank of sample in each assay belonging to the corresponding assay format or format variant group. Control samples are sorted on the horizontal axis according to their median rank as calculated across all IASP2018 assays. The global median rank of controls in 2018 is shown as a solid black line.

ESM Figure 11

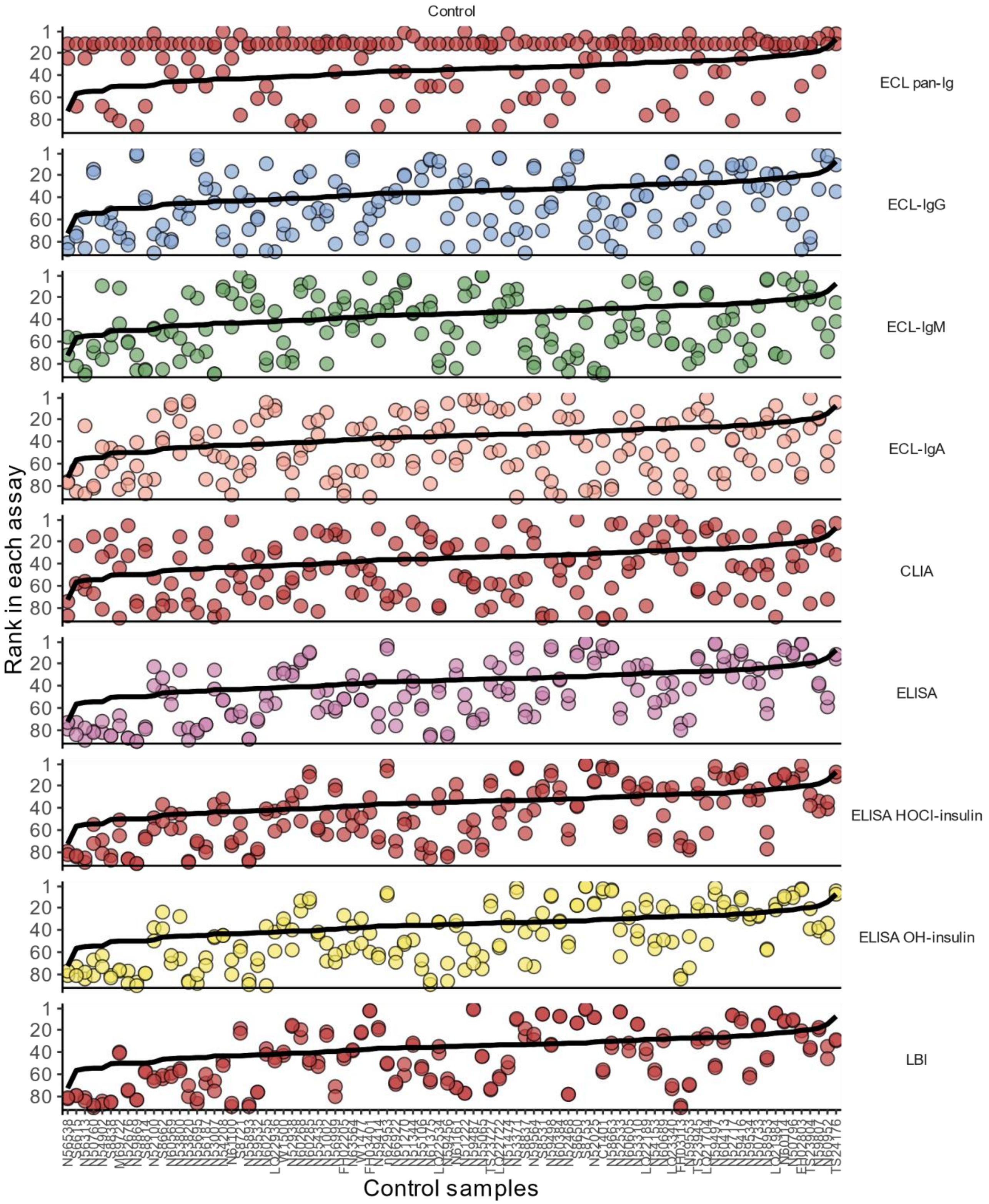

ESM figure 11. Rankings of IAA results in control samples included in IASP2018 (ECL, CLIA, ELISA and LBI assays).

Circles show the rank of sample in each assay belonging to the corresponding assay format or format variant group. Control samples are sorted on the horizontal axis according to their median rank as calculated across all IASP2018 assays. The global median rank of controls in 2018 is shown as a solid black line.

ESM Figure 12

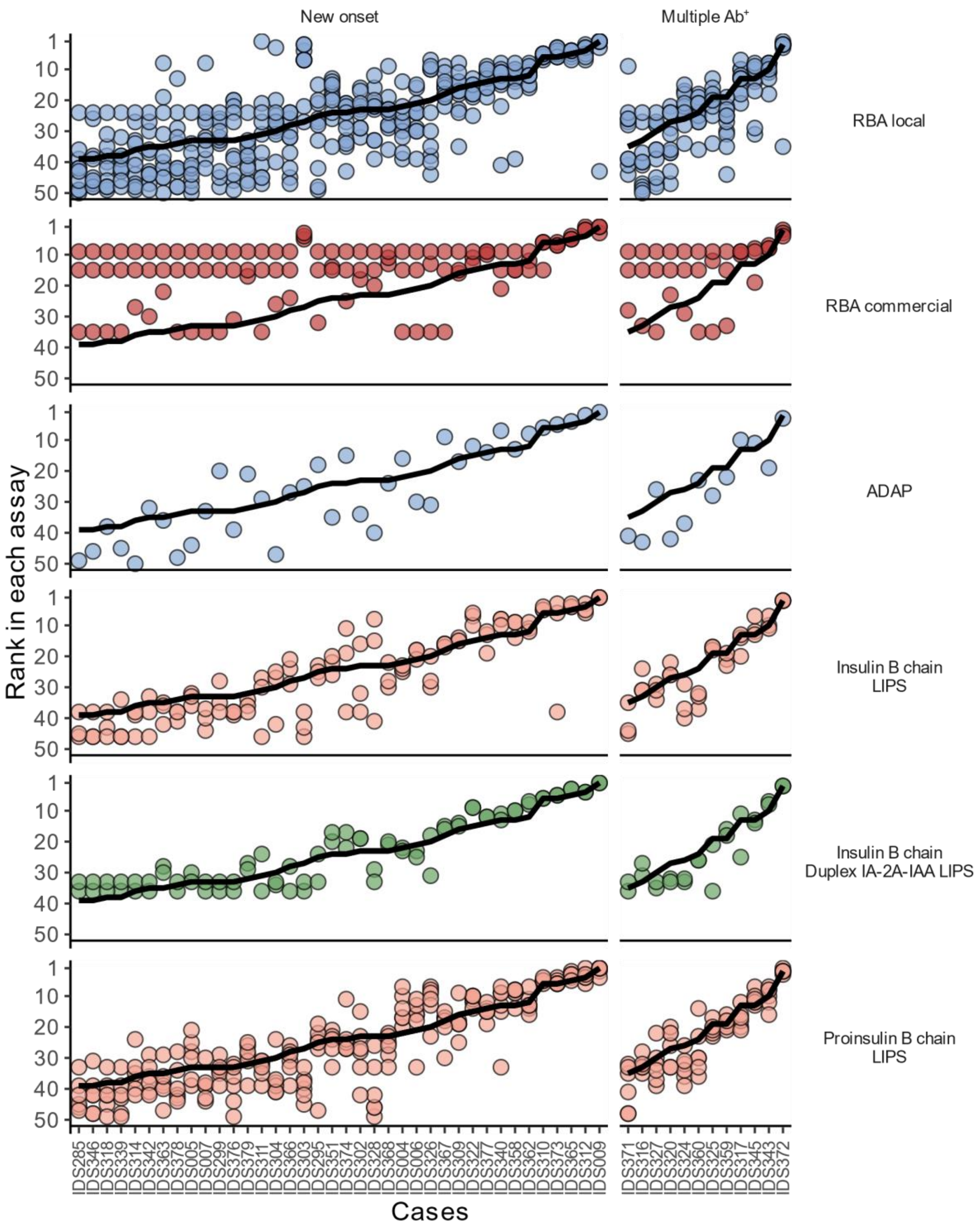

ESM figure 12. Rankings of IAA results in case samples included in IASP2020 (RBA, LIPS and ADAP assays).

Circles show the rank of sample in each assay belonging to the corresponding assay format or format variant group. Samples are grouped into new onset T1D and multiple autoantibody positive and within each group are sorted on the horizontal axis according to their median rank as calculated across all IASP2020 assays. The global median rank of cases in 2020 is shown as a solid black line.

ESM Figure 13

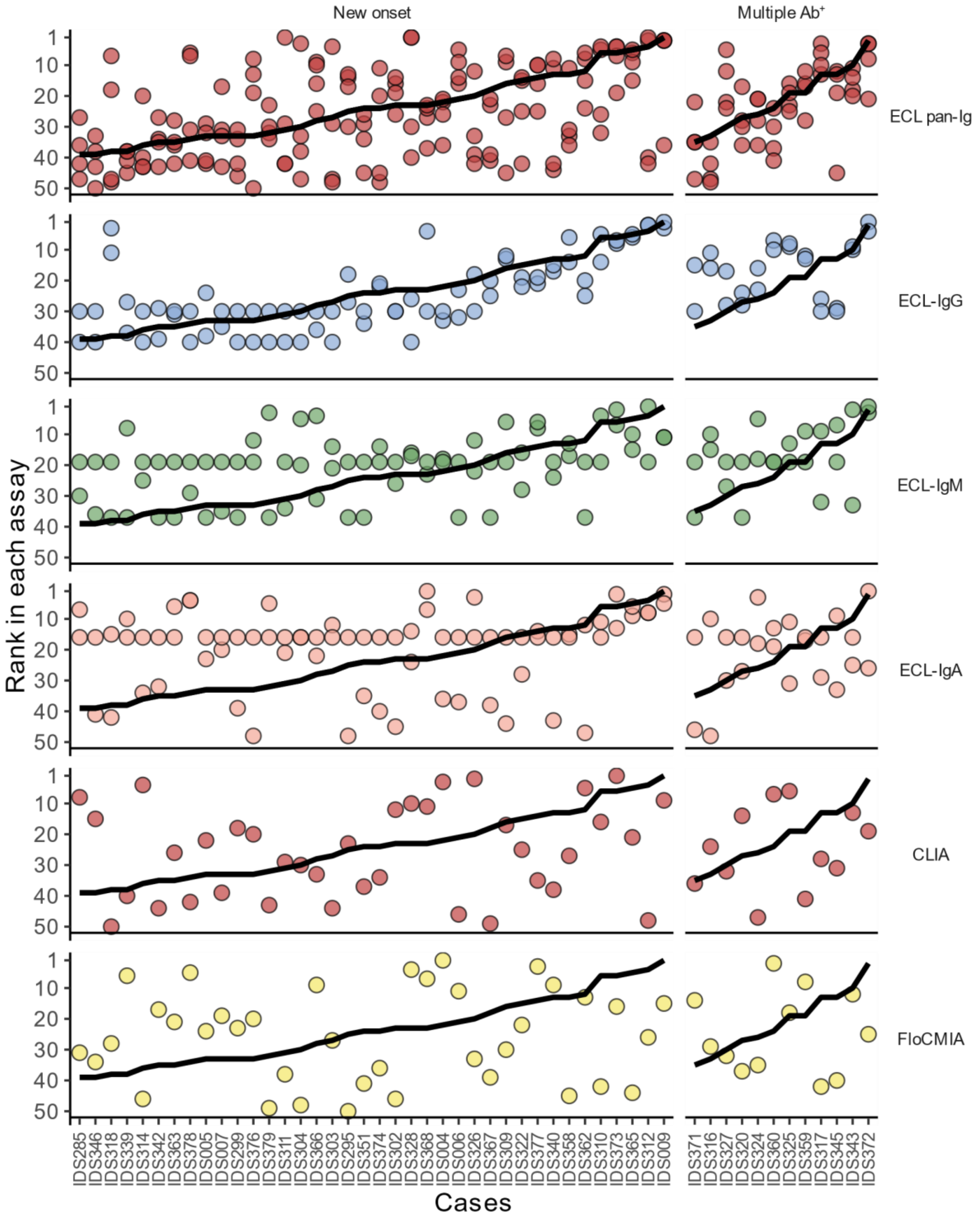

ESM figure 13. Rankings of IAA results in case samples included in IASP2020 (ECL, CLIA and FloCMIA assays).

Circles show the rank of sample in each assay belonging to the corresponding assay format or format variant group. Samples are grouped into new onset T1D and multiple autoantibody positive and within each group are sorted on the horizontal axis according to their median rank as calculated across all IASP2020 assays. The global median rank of cases in 2020 is shown as a solid black line.

ESM Figure 14

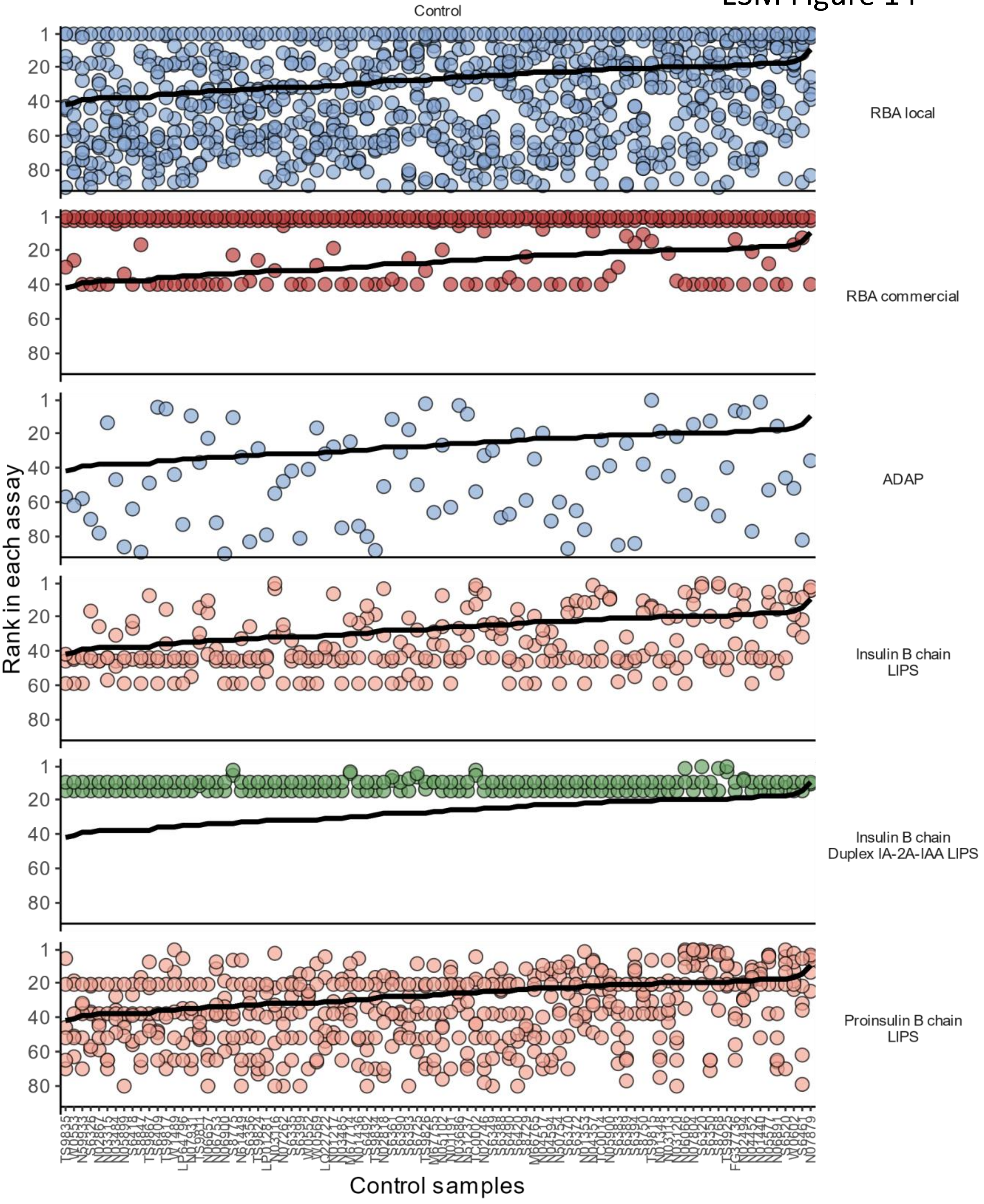

ESM figure 14. Rankings of IAA results in control samples included in IASP2020 (RBA, LIPS and ADAP assays).
Circles show the rank of sample in each assay belonging to the corresponding assay format or format variant group. Control samples are sorted on the horizontal axis according to their median rank as calculated across all IASP2020 assays. The global median rank of controls in 2020 is shown as a solid black line.

ESM Figure 15

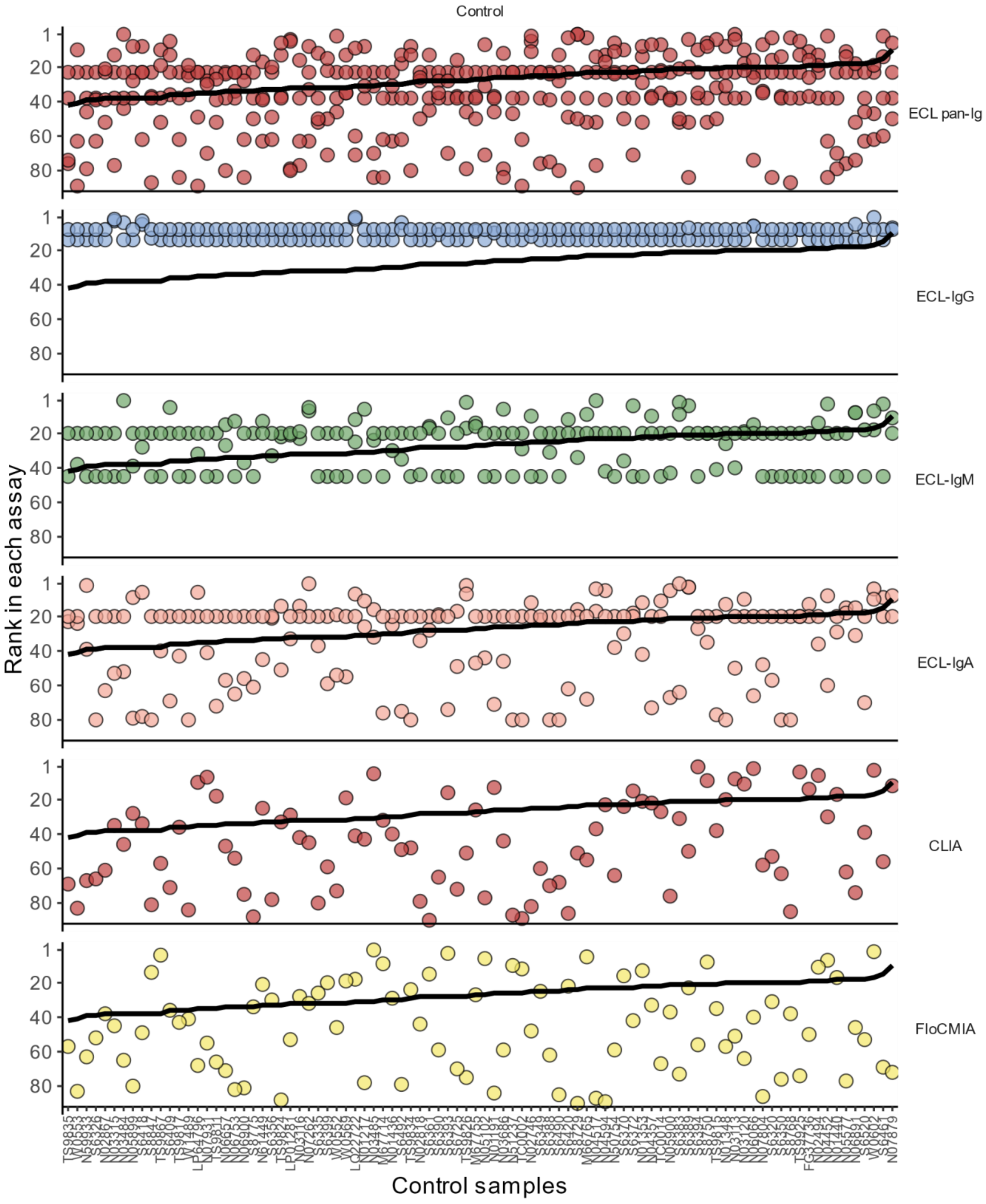

ESM figure 15. Rankings of IAA results in control samples included in IASP2020 (ECL, CLIA and FloCMIA assays).
Circles show the rank of sample in each assay belonging to the corresponding assay format or format variant group. Control samples are sorted on the horizontal axis according to their median rank as calculated across all IASP2020 assays. The global median rank of controls in 2020 is shown as a solid black line.

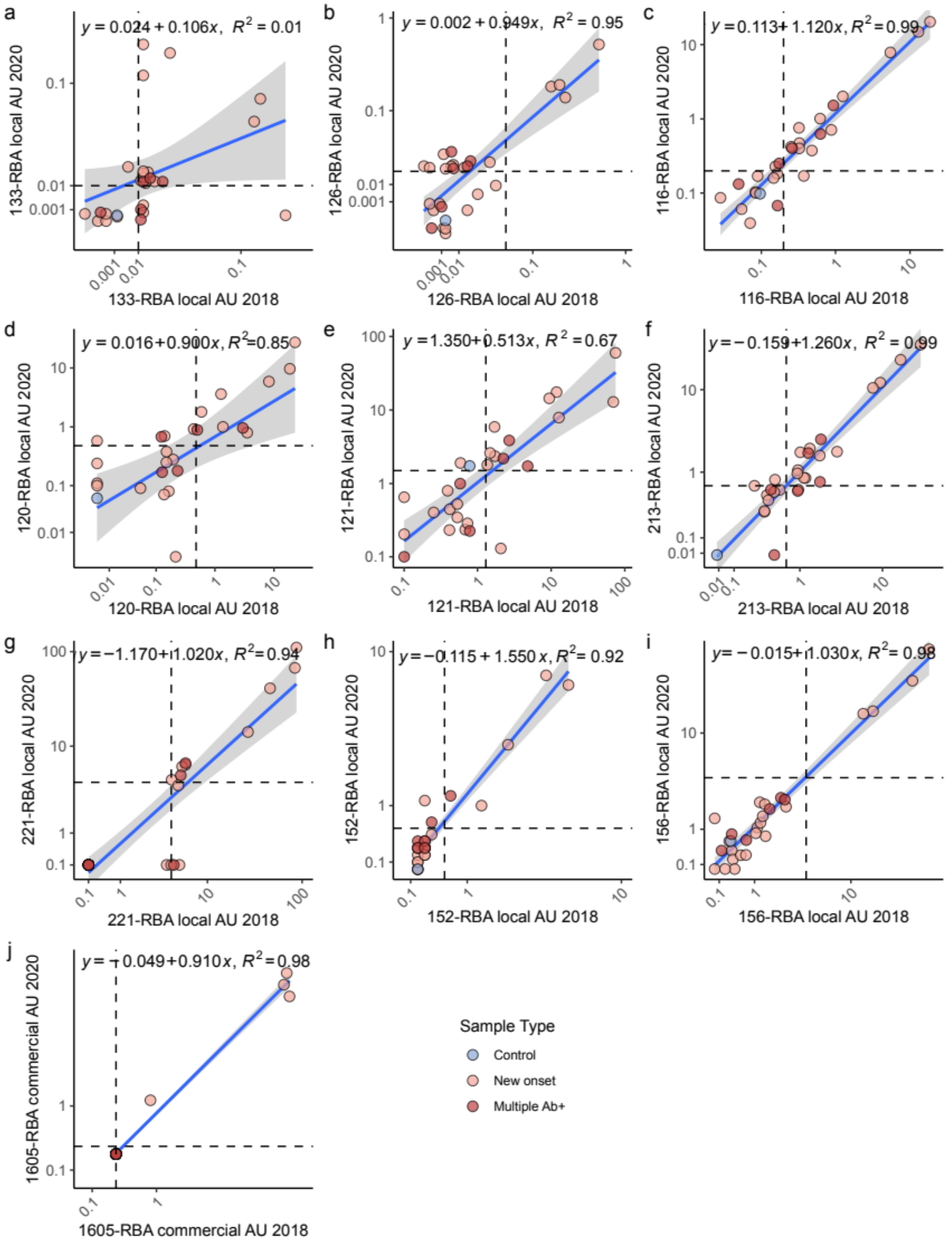

ESM Figure 16. Correlation of local arbitrary units in IASP2018 and IASP2020. Scatterplot of local RBA (panels a-i) and commercial RBA (panel j) assay results submitted in IASP2018 and IASP2020 for serum samples distributed in both workshops (n=28). Circles represent the local units assigned to each sample, fill color correspond to the indicated sample type. On each panel are shown the regression line (blue), its 95% CI (grey area), and the corresponding linear regression equation and coefficient, calculated on a linear scale data. Vertical and horizontal dashed lines correspond to the reported positivity thresholds.

ESM Figure 17

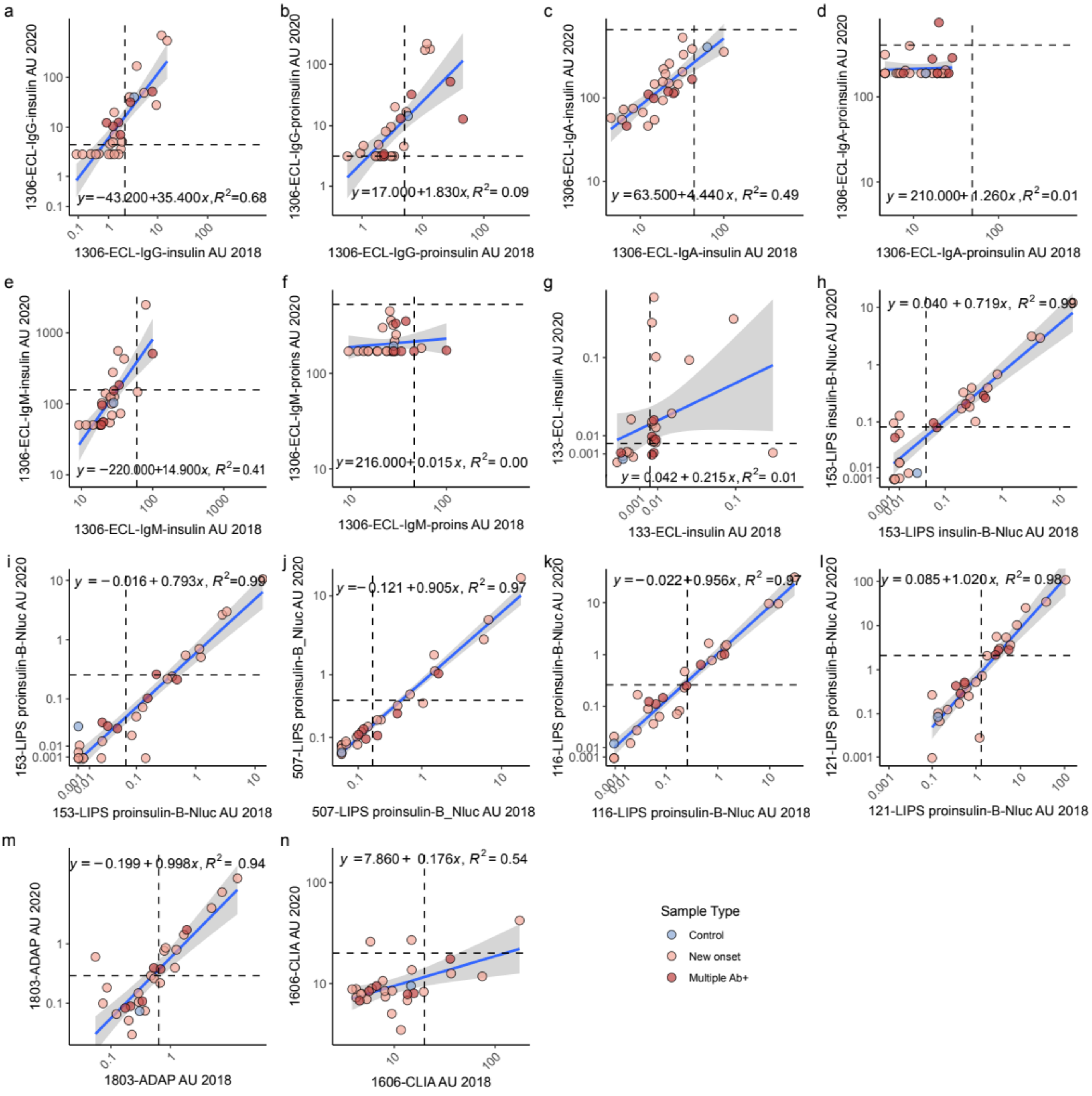

ESM Figure 17. Correlation of local arbitrary units in IASP2018 and IASP2020. Scatterplot of ECL (panels a-g), LIPS (panels h-l), ADAP (panel m), and CLIA (panel n) assays results submitted in IASP2018 and IASP2020 for serum samples distributed in both workshops (n=28). Circles represent the local units assigned to each sample, fill color correspond to the indicated sample type. On each panel are shown the regression line (blue), its 95% CI (grey area), and the corresponding linear regression equation and coefficient, calculated on a linear scale data. Vertical and horizontal dashed lines corresponds to the reported positivity threshold.

ESM Figure 18

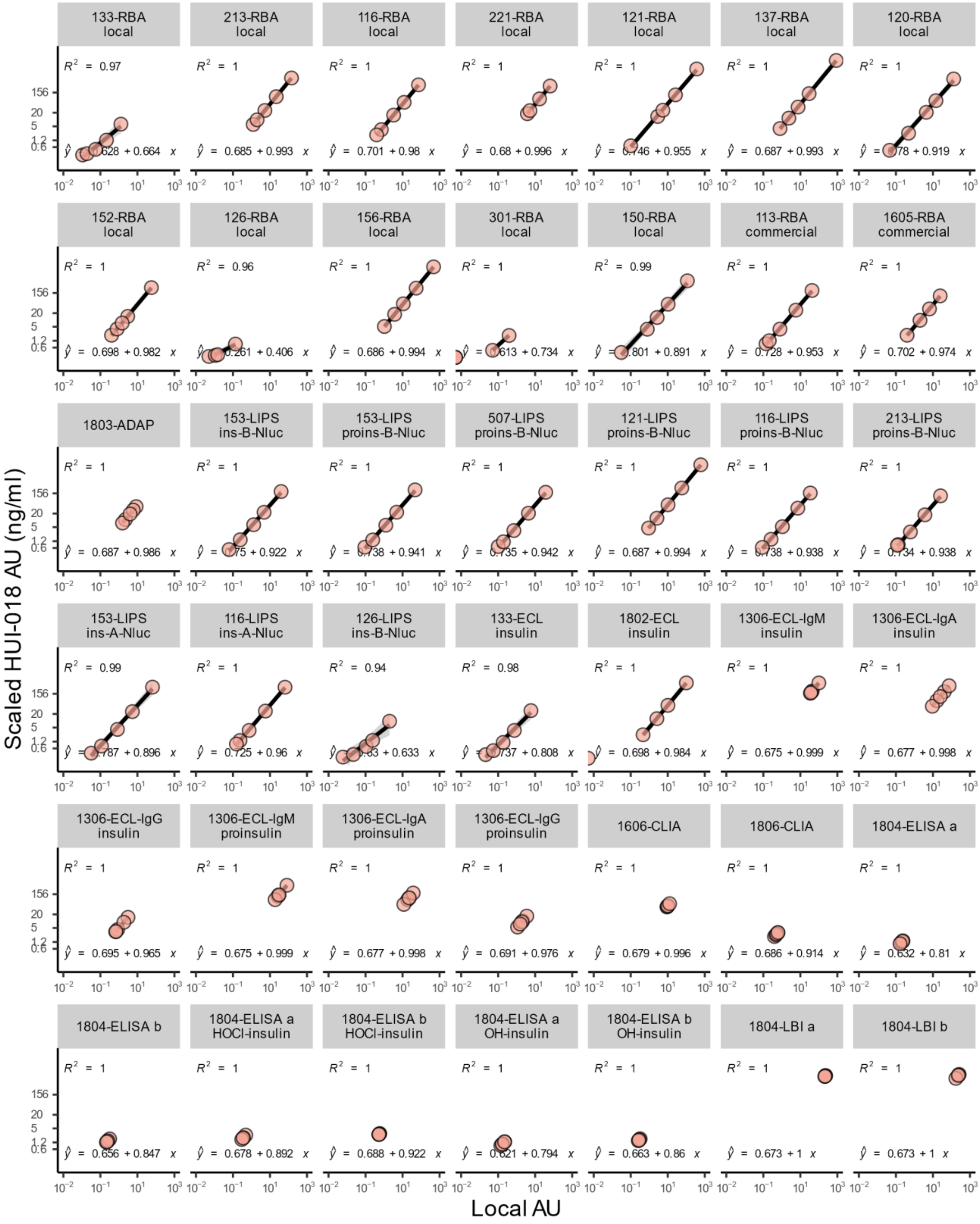

ESM figure 18. Correlation of laboratory assigned local units with scaled HUI-018 units in HUI-018 dilutions included in the IASP2018 set.
Orange circles indicate single IAA measurements in HUI-018 dilutions (0.6, 1.2, 5, 20, 156 ng/ml). The laboratory ID and the assay format and format variant are reported on each panel. Shown are the regression line (solid black), the regression equation and the R^2 coefficient.

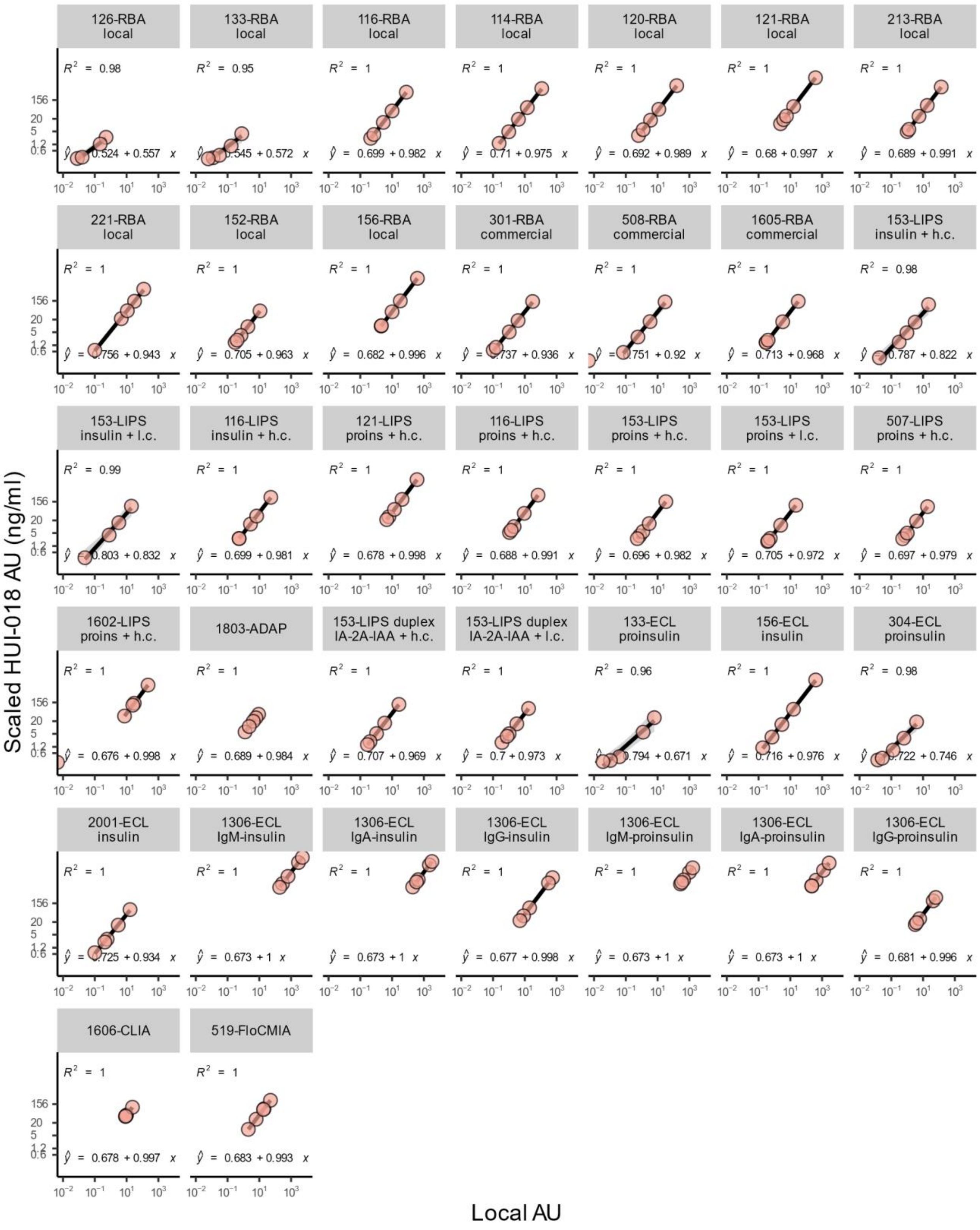

ESM figure 19. Correlation of laboratory assigned local units with scaled HUI-018 units in HUI-018 dilutions included in the IASP2020 set.

Orange circles indicate single IAA measurements in HUI-018 dilutions (0.6, 1.2, 5, 20, 156 ng/ml). The laboratory ID and the assay format and format variant are reported on each panel. Shown are the regression line (solid black), the regression equation and the R^2 coefficient.

ESM Figure 20

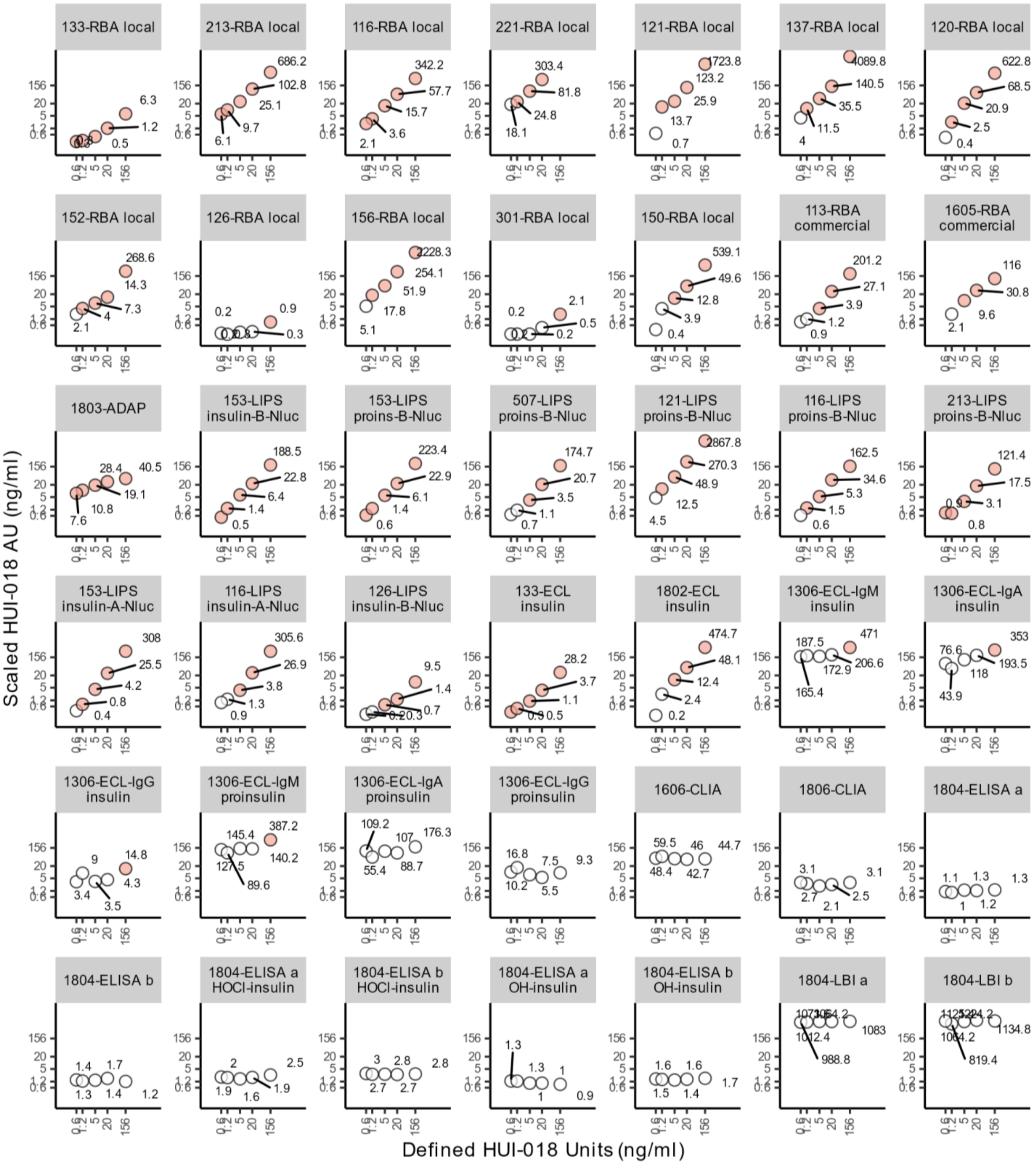

ESM figure 20. Scatterplot of HUI-018 mAb dilutions concentration vs measured concentration in IASP2018.

The laboratory ID and the assay format and format variant are reported on each panel. Circles show the local units rescaled using the HUI-018 standard curve in relation to the expected concentration of each the HUI-018 mAb dilution (0.6, 1.2, 5, 20, 156 ng/ml). Circle fill stands for a positive (orange) or negative (white) score.

ESM Figure 21

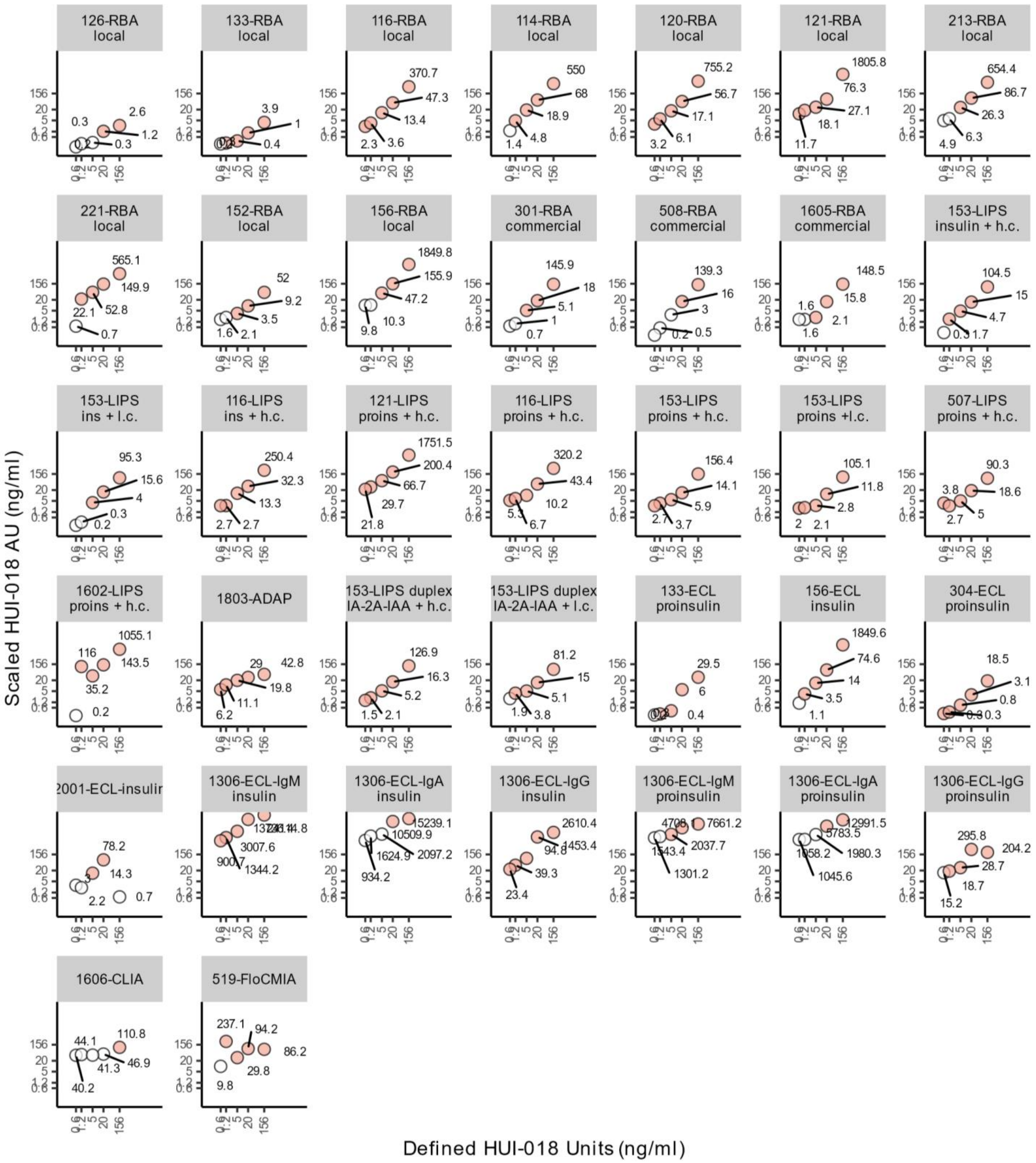

ESM figure 21. Scatterplot of HUI-018 mAb dilutions concentration vs measured concentration in IASP2020.

The laboratory ID and the assay format and format variant are reported on each panel. Circles show the local units rescaled using the HUI-018 standard curve in relation to the expected concentration of each the HUI-018 mAb dilution (0.6, 1.2, 5, 20, 156 ng/ml). Circle fill stands for a positive (orange) or negative (white) score.

ESM Figure 22

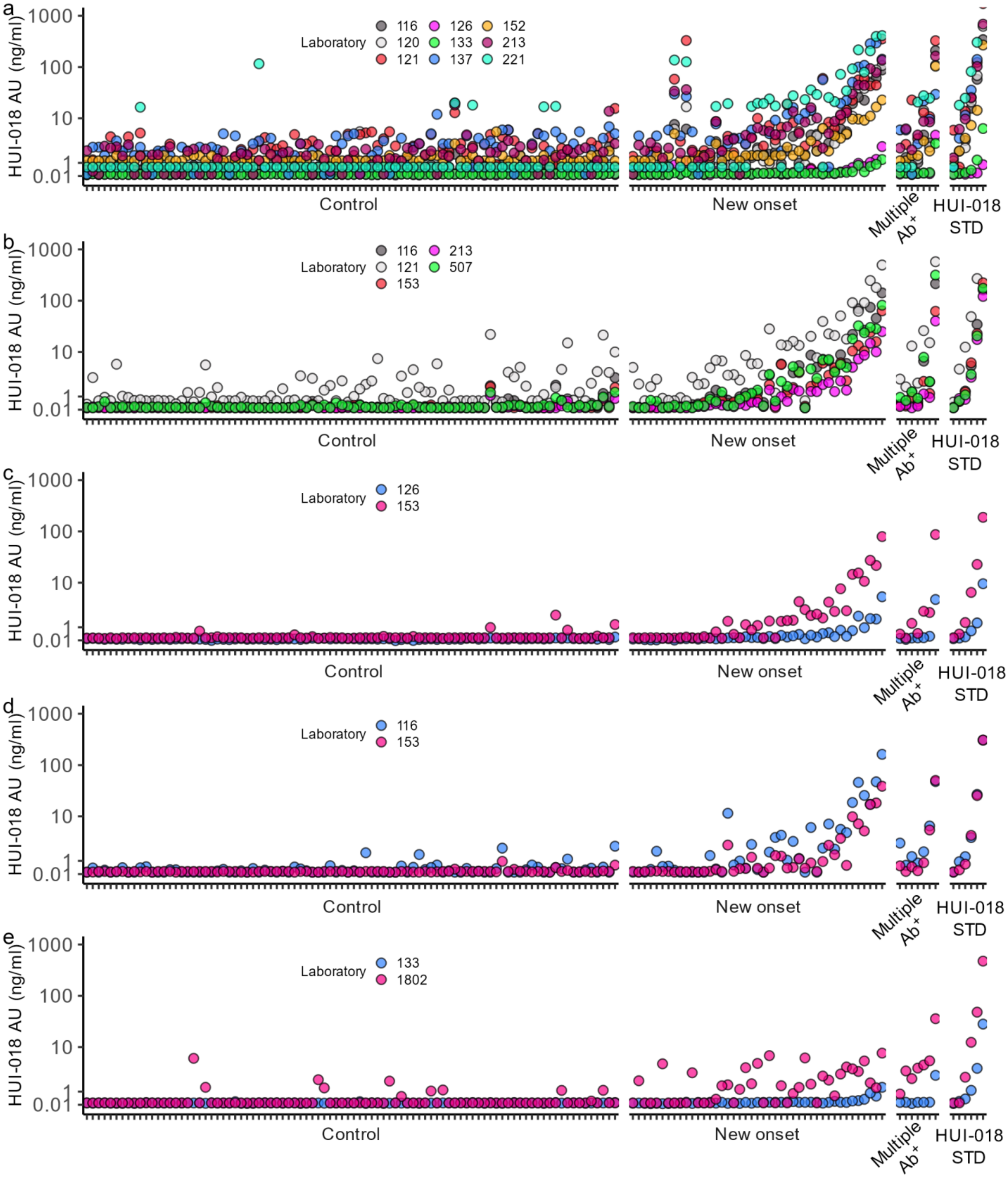

ESM figure 22. Stripcharts of common HUI-018 units in different assay formats submitted to the IASP2018. Shown assays were selected according to good performance (pAUC95>0.015) and adoption of a detection system compatible with the measurement of a mouse IgG mAb. Panels group assays according to format and format variant: RBA local (a), LIPS proinsulin-B-Nluc (b), LIPS insulin-B-Nluc (c), LIPS insulin-A-Nluc (d), ECL pan Ig (e). Samples are sorted horizontally into groups (blood donor controls, new onset T1D, multiple autoantibody positive FDR, and HUI-018 standards) and within each group according to their median rank as calculated across all IASP2018 assays. Circles show individual sample results in common HUI-018 units; circle fill represents individual assays as shown in the legend.

ESM Figure 23

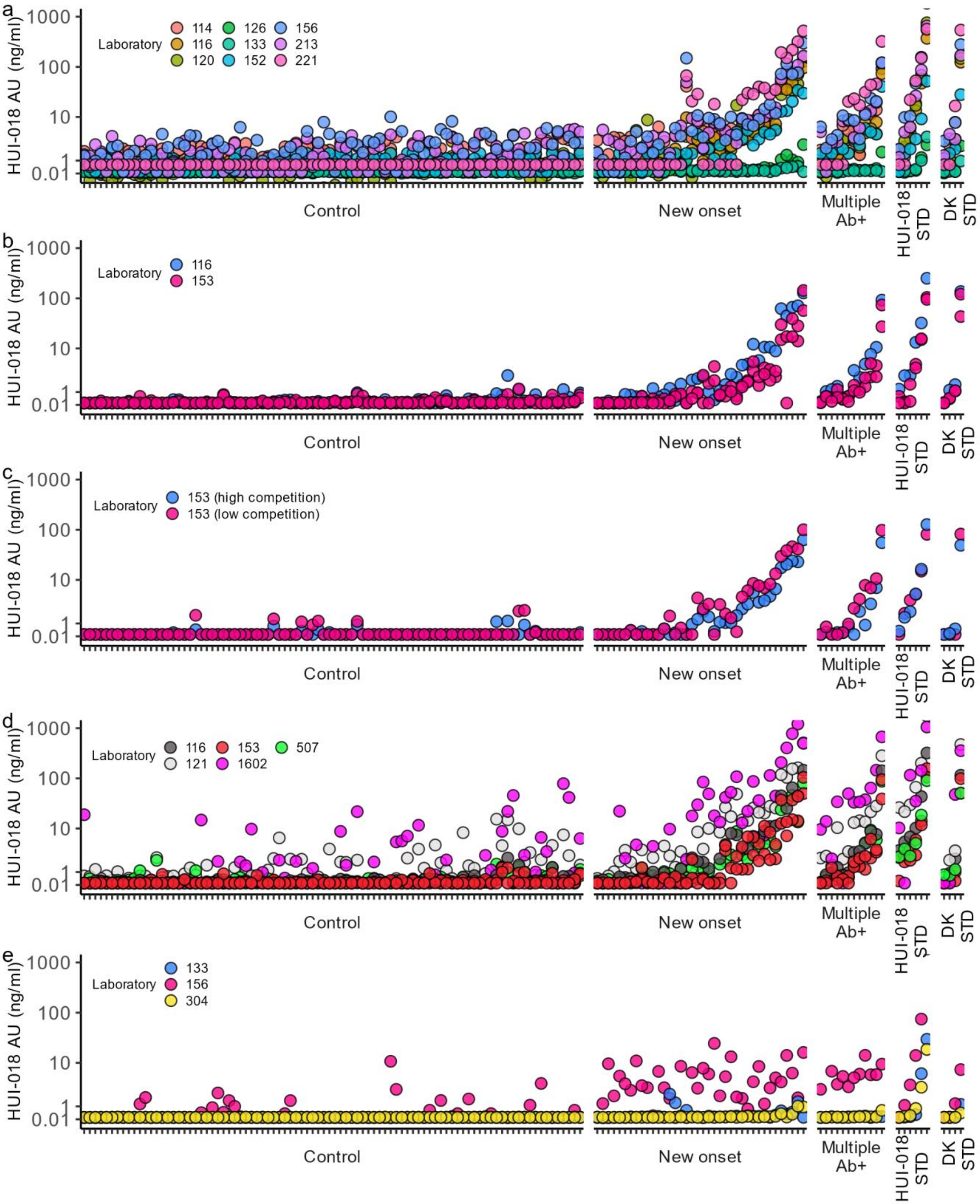

ESM figure 23. Stripcharts of common HUI-018 units in different assay formats submitted to the IASP2020. Shown assays were selected according to good performance (pAUC95>0.015) and adoption of a detection system compatible with the measurement of a mouse IgG mAb. Panels group assays according to format and format variant: RBA local (a), LIPS insulin-B-Nluc (b), LIPS Duplex IA-2A-IAA (c), LIPS proinsulin-B-Nluc (d), ECL pan Ig (e). Samples are sorted horizontally into groups (blood donor controls, new onset T1D, multiple autoantibody positive FDR, DK and HUI-018 standards dilutions) and within each group according to their median rank as calculated across all IASP2020 assays. Circles show individual sample results in common HUI-018 units; circle fill represents individual assays as shown in the legend.
